# Spiropyran/Merocyanine Amphiphile in Various Solvents: A Joint Experimental–Theoretical Approach to Photophysical Properties and Self-Assembly

**DOI:** 10.3390/ijms231911535

**Published:** 2022-09-29

**Authors:** Vladyslav Savchenko, Nino Lomadze, Svetlana Santer, Olga Guskova

**Affiliations:** 1 Institute Theory of Polymers, Leibniz Institute of Polymer Research Dresden, Hohe Str. 6, 01069 Dresden, Germany; 2 Institute of Physics and Astronomy, University of Potsdam, Karl-Liebknecht-Strasse 24-25, 14476 Potsdam, Germany; 3 Dresden Center for Computational Materials Science (DCMS), Technische Universität Dresden, 01062 Dresden, Germany

**Keywords:** spiropyran/merocyanine isomerization, negative photochromism, time-resolved UV-vis measurements, molecular modeling

## Abstract

This joint experimental-theoretical work focuses on molecular and photophysical properties of the spiropyran-containing amphiphilic molecule in organic and aqueous solutions. Being dissolved in tested organic solvents, the system demonstrates positive photochromism, i.e., upon UV stimulus the colorless spiropyran form is transformed into colorful merocyanine isomer. However, the aqueous solution of the amphiphile possesses a negative photochromism: the orange-red merocyanine form becomes thermodynamically more stable in water, and both UV and vis stimuli lead to the partial or complete photobleaching of the solution. The explanation of this phenomenon is given on the basis of density functional theory calculations and classical modeling including thermodynamic integration. The simulations reveal that stabilization of merocyanine in water proceeds with the energy of ca. 70 kJ mol${}^{-1}$, and that the Helmholtz free energy of hydration of merocyanine form is 100 kJ mol${}^{-1}$ lower as compared to the behavior of SP isomer in water. The explanation of such a difference lies in the molecular properties of the merocyanine: after ring-opening reaction this molecule transforms into a zwitterionic form, as evidenced by the electrostatic potential plotted around the opened form. The presence of three charged groups on the periphery of a flat conjugated backbone stimulates the self-assembly of merocyanine molecules in water, ending up with the formation of elongated associates with stack-like building blocks, as shown in molecular dynamics simulations of the aqueous solution with the concentration above critical micelle concentration. Our quantitative evaluation of the hydrophilicity switching in spiropyran/merocyanine containing surfactants may prompt the search for new systems, including colloidal and polymeric ones, aiming at remote tuning of their morphology, which could give new promising shapes and patterns for the needs of modern nanotechnology.

## 1. Introduction

A diversity of stimuli-responsive synthetic amphiphiles have been elaborated to develop supramolecular self-assemblies whose structures can be controlled by external stimuli, e.g., pH, heat, and light. Light as a contactless stimulus offers multiple advantages such as tunable optical wavelength and intensities as well as high temporal and spatial control. Several classes of photoresponsive amphiphilic molecules are distinguished: intrinsic light-switches, photoresponsive molecular amphiphiles (a photoresponsive unit is chemically included into a classical surfactant “head-tail” structure), and photoresponsive supramolecular amphiphiles (a photoresponsive component is introduced via noncovalent interactions) [1]. The self-organized ensembles of photoresponsive amphiphiles manipulated by light fundamentally enable new pathways towards aqueous bio-compatible materials, that can change certain properties in a predictable manner or can provide best performance when operation circumstances change [2].

The moieties that are utilized to render amphiphiles the photoresponsive nature are azobenzene [3,4,5,6], stilbene [7], dithienylethene [8] and spiropyran [9,10,11], to name a few [2]. Such amphiphiles have exhibited, for example, (i) smart principles for the dynamic control of surface tension of aqueous solutions [12], (ii) phenomenon of the light-driven diffusioosmosis upon which one can remove, gather or pattern a particle assembly at a solid-liquid interface [13], (iii) the photo-controlled stability and breakage of foams [14], (iv) new macroscopic functions such as liquid droplet transport [15] and bubble manipulation [9], (v) photoswitchable catalytic properties [16], (vi) photochemical OFF/ON cytotoxicity switching [17], etc.

The spiropyran/merocyanine-containing (SP/MC) amphiphiles have also been of great interest, e.g., for a controllable change of the physicochemical properties of the micellar solution as a consequence of photoisomerization [18,19,20,21], for the construction of the reversible shape and color-changing block copolymer particles [22], for the spatiotemporal control of bubble-propelled micromotors [23], as photoinitiators in polymerizations [24].

**Figure 1:**
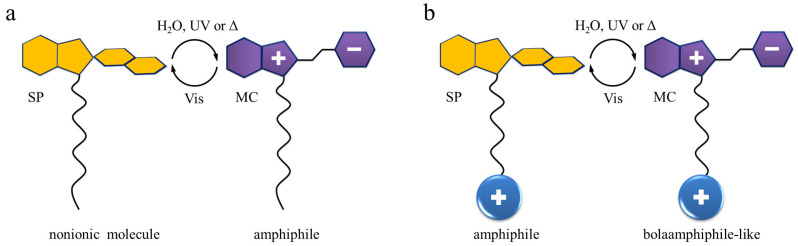
Schematic representation of the surfactants and their mutual interconversion under various stimuli: SP/MC is considered as a head (**a**) or as a part of the tail (**b**). We underline here different properties of the molecules prior and after isomerization. More details can be found in the main text.

According to the location of the SP/MC unit in the amphiphile structure, the molecules can be classified as the following: The first group represents surfactants with spiropyran/ merocyanine introduced as the head group (Figure 1a) [9,17,23,24,25,26,27,28,29,30,31,32,33,34,35,36]. For the second one, SP/MC is a part of the tail of a photoresponsive amphiphile included at its different positions: at the end of the hydrocarbon chain [11,18,19,20,22,37,38,39,40,41,42,43,44] (Figure 1b) or in its middle [10].

The photoisomerization causes a dramatic change of the hydrophilic–hydrophobic balance of the amphiphile: apparently, MC itself is a zwitterionic form and is more hydrophilic than the nonionic SP state, as shown in experiments [18,36]. In addition, there are two interesting aspects, we would like to discuss. For the first group mentioned in the classification, where SP/MC is the head of the surfactant, both UV stimulus and/or insertion into water will turn a nonpolar molecule into a classical surfactant structure. For the amphiphiles with SP placed in a tail, the aforementioned triggers transform a classical surfactant with a positively [18,19,20,22,37] or negatively [14,37,45,46] charged head group, i.e.,–N(CH${}_{3}$)${}_{3}$${}^{+}$ or –SO${}_{3}$${}^{2-}$, respectively, and a hydrophobic SP-containing a tail into a structure resembling a bolaamphiphile, i.e., an amphiphilic molecule that has hydrophilic groups at both ends of a sufficiently long hydrophobic hydrocarbon chain [18,47]. Consequently, the length of the alkyl could be another important parameter of the surface activity.

The shortest hydrophobic hydrocarbon chain between SP and a permanently charged headgroup known from the literature is propyl (C3) [11,37,39,40,41,42,43,44,48]. Amphiphiles with butyl-linked (C4) SP and headgroup were studied by Braunschweig et al. [14]. The surfactants with hexyl (C6) chain were the object of experiments of Sakai et al. [19,20] and Gan et al. [38]. The surfactants with the longest dodecyl (C12) chain were synthesized by Sakai et al. [20] and Kim et al. [22]. Only the latter example could probably self-assemble in a bolaamphiphile way, that is to demonstrate a rich self-assembly behavior.

**Figure 2:**
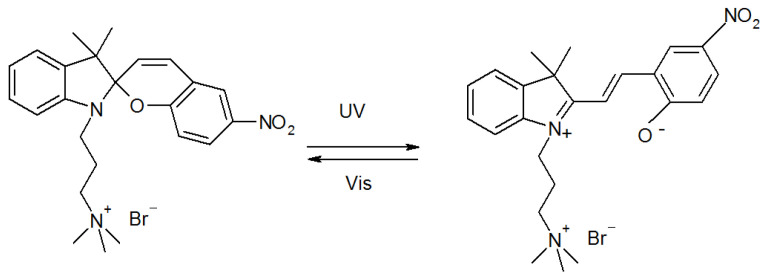
Spiropyran/merocyanine photochromic amphiphilic couple studied in this paper. This surfactant contains a hydrophobic propyl (C3) hydrocarbon chain terminated with a permanently charged quaternary ammonium headgroup and introduced in the ω-position of spiropyran (**left**) bearing an –NO${}_{2}$ substituent in para-position to the pyran oxygen. It was reported that the electron-acceptor substituents in para-position stabilize the zwitterionic MC form (**right**) and drastically lower the p*K*${}_{a}$ [11,62].

Theoretical studies published so far include quantum chemical description of the photoinduced ring-opening reaction of spiropyrans bearing various substituents [49,50,51,52,53,54,55], classical, semiclassical and ab initio calculations of the hydration mechanisms and the water shells around spiropyran and merocyanine, as well as MC protonated forms [49,56,57,58,59], and theoretical characterizations of the optical properties [60]. To the best of our knowledge, there are only a few papers devoted to the theory and modeling of the SP/MC amphiphiles. In 2004, Bae and Arnold have used an empirical extension of the continuum model to reproduce the absorption spectrum of the optical probe merocyanine 540 (M540, surfactant with permanently charged -SO${}_{3}$ head and a tail consisting of a propyl segment and MC) in various solvents with the aim to determine dielectric constant and refractive index of the bulk solvent [61]. The findings indicated that specific solute-solvent interactions, which are ignored in a continuum model are constant or even negligible for all the studied solvents and their mixtures, with the exception of water. Hammarson et al. in 2013 have characterized six water-soluble spiropyran derivatives in water over a broad pH-interval [11]. For one of them, which is the object of our paper (Figure 2), the authors have modeled the hydrolysis mechanism of merocyanine in an acidic environment using the density functional approach with implicit and explicit water content. They have found that only nonprotonated MC isomer undergoes hydrolysis, and therefore, the hydrolytic degradation is halted at pH values where the protonated MC is the dominating open form. The noncovalent interactions (NCI) in dimers of a surfactant having a triazole linker connecting the MC polar head to the octyloxy hydrophobic chains have been identified by Zhang and co-authors [35] in density functional theory (DFT) calculations. The NCI plot indicated the presence of the stacking interactions between the aromatic rings and the hydrophobic interplay of the alkyl chains, which explained the origin of H-aggregates in methanol solution of various concentrations, detected experimentally only for the MC form and absent for the SP form. In 2021, Aldaz et al. [48] simulated the spiropyran-based photoacid with charged -SO${}_{3}$ head separated by a short C3 alkyl from the SP part. For most calculations the propyl sulfonate group of a photoacid was truncated to a methyl group to facilitate geometry optimisation. Very recently, Reifarth and others [10] investigated switching behavior and remote control over emulsion stability of a dual pH and light-responsive SP surfactant with the photochrome segment included in the middle of the hydrophobic tail. Again, the density functional theory was utilized for prediction of the interconversion mechanism and the energy barriers of the mechanism’s stages, which are in fair agreement with experimental value of the activation energy.

In the current study, we investigate photoresponsive behaviour of the spiropyran-modified cationic surfactant in different solvents such as chloroform, ethanol, acetonitrile, dimethyl sulfoxide and water both experimentally and theoretically, combining a number of quantum-chemical and classical simulation techniques. As mentioned above, the photoresponsive amphiphiles have been previously simulated as isolated molecules in water by the use of dielectric background or considering one water molecule as one of the reactants in the hydrolysis reaction. To the best of our knowledge, no studies have been conducted to investigate the photoresponse of the SP/MC amphiphile in a number of solvents. From this point of view, our originality lies in the description of the molecular properties of the SP and MC forms with a special emphasis on their self-assembly in water as well as on the supramolecular structures they are building being modeled in al-atom molecular dynamics (MD) simulations in water. On the basis of a similar concept, Eilmes [58] has reported the electrostatic potentials, the static dipole moments of spiropyrans and the hydrogen bonding with water, i.e., the properties, that are important for the anticipation of a possible behavior in a watery environment; however, this research topic still requires more detailed study not only from a qualitative standpoint (e.g., by considering larger systems and a direct comparison with experimental data) but also from a quantitative one. In our current work, systematic studies have been carried out as follows: (i) synthesis and theoretical description of the molecular properties of the amphiphile; (ii) determining the photophysical properties in the chloroform, ethanol, acetonitrile, dimethyl sulfoxide, and water; (iii) calculation of the solvation free energies of the surfactants in these solvents using thermodynamic integration (TI), and (iv) characterizing the aqueous solution self-assembly properties of the SP/MC surfactant, assessed by MD simulation. These measurements and simulations were performed both before and after UV/visible light irradiation to evaluate the photoresponsive behavior.

## 2. Results

### 2.1. Molecular Properties

**Figure 3:**
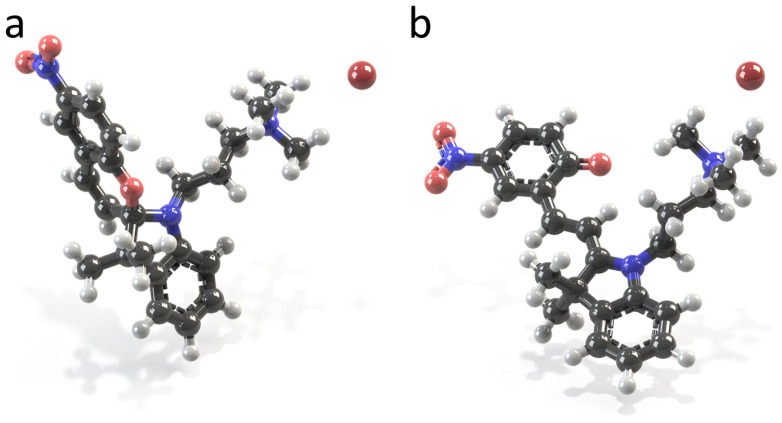
Snapshots of spiropyran ((**a**), TMAB-C3-SP) and merocyanine ((**b**), TMAB-C3-MC) surfactants with Br${}^{-}$ anions. The structures are as received after geometry optimization in implicit water. Carbon, oxygen, nitrogen, hydrogen and bromine are shown as grey, red, blue, white and burgundy spheres, respectively. The Br${}^{-}$ anion is in the vicinity of trimethylammonium head, symmetrically centred between three methyl groups of the TMA with the distance N${}^{+}$ – Br${}^{-}$ of 4.23 ± 0.06 Å on average. The abbreviations TMAB, C3, and SP/MC stand for trimethylammonium bromide, propyl chain, and spiropyran/merocyanine molecular segment, respectively. The multiplicity of the chemical bonds is not shown here, except for both benzene rings and nitro groups. Cartesian coordinates of both isomers are provided in Supplementary Information.

Characterization of the molecular properties is opened with a discussion of the isomer geometries of the amphiphiles in water and their thermodynamic stability. Two snapshots of the optimized geometries of spiropyran TMAB-C3-SP and merocyanine TMAB-C3-MC are shown in Figure 3. For both SP and MC, several geometries belonging to local minima on the potential energy surface can be distinguished, for example, two closed-form structures, differing by spatial arrangement of the O atom with respect to the indoline moiety, two open-form structures, differing by rotation of the benzopyran around the adjacent C=C bond, as noticed by some authors [10,50,52,53], the open forms with cisoid or transoid C=C double bond [63], or additional pairs of structures due to the flexibility of the substituents, e.g., as discussed by Eilmes [58]. Analysis of the published data has shown that the energy difference between the corresponding structural isomers is not crucial and that the most essential distinction corresponds to the cisoid and transoid form of the merocyanines (our estimation for the energy difference between the transoid and the cisoid forms of the TMAB-C3-MC in vacuum is ca. +5.59 kJ mol${}^{-1}$, that is the cis TMAB-C3-MC is energetically less favourable). At the same time, the cisoid form is considered as a transition state in the isomerisation reaction pathways [10,48,64]. Since the investigation of such mechanisms goes beyond the scope of this paper, we explore properties of two structures depicted in Figure 3.

We should also note that different initial placements of Br counterions have been simulated: not only the symmetrical position with respect to three methyl groups of TMA head, as shown in Figure 3, but also bromide in-between two nitrogen atoms along the C3 alkyl chain, and in-between TMA group and the phenyl ring. The most energetically favourable position of Br${}^{-}$ for both isomers is the first one, shown in Figure 3. Similar results are described by other authors for various amphiphiles with TMAB head [3,65]. All other calculated samples are with the energy difference exceeding tens of kJ mol${}^{-1}$ as compared to the reference system.

The values of bond lengths, angles, and dihedrals are dependent on the substituents present in SP and MC, as shown by Seiler et al. in the experimental studies of spiropyrans and their polymorphs [66]. For example, the C${}^{spiro}$ –O bond has a length of 1.453 Å for SP illustrated in Figure 3a. The same bond is 1.496 Å in experimental studies by Zhang et al. [35], 1.456 Å in measurements described by Pugachev et al. [67] and varies from 1.4558 to 1.488 Å, as reported by Seiler and co-workers [66]. The chemical bond C${}^{spiro}$–N is 1.472 Å; the experimental values are 1.438 [35], 1.452 [67] and from 1.426 to 1.446 Å [66]. The values reported in theoretical calculations published in the literature [49,53,54,56,57] are closer to the mentioned values evaluated in this work. For example, for MC (Figure 3b) the length of the double bond formed upon photoisomerization due to the ring opening is 1.385 Å, which coincides with the value reported by Murugan et al. [56] in Car-Parrinello MD of MC and SP in water and by Sheng et al. [49] for a set of compounds bearing various substituents and immersed in different solvents.

The spiropyran in the conventional SP/MC pairs is commonly a more favorable state [35,49,52,59,64]. Under UV light it converts to the thermodynamically less stable state (MC) which is a more deeply colored form, and similar systems are called positive photochromic systems [68]. However, the data in Table 1 clearly reveal that the merocyanine isomer represents a thermodynamically more stable form, as compared to the spiro-compound. There is a few spiropyrans known from the literature, especially those with OH, COOH, NH${}_{2}$, and NR${}_{3}$ groups exhibiting the so-called negative photochromism [18,68,69,70,71]. Thus, the deeply-colored MC solution spontaneously formed by mixing a photochrome with a polar solvent is photo-bleached under visible light. One reason for a more stable MC form in polar solvents is the stabilization of the phenolate anion of the MC form by the introduction of the strong acceptor substituents, like nitro, carboxy groups; or specific interactions with the solvent molecules, including preferential solvation; the formation of the hydrogen bonds with OH, COOH and other groups. The negative photochromism can be also promoted if SP/MC is introduced into polymer matrix, conjuncted with some macromolecules or builds complexes with metal ions [69]. The photophysical properties are discussed in details below.

The energy differences of the MC and the SP states also suggest that one could expect the spontaneous ring-opening reaction upon dissolution in a solvent. As mentioned in some publications [68,69], the solvents of higher polarity, i.e., with the largest values of the dielectric permittivity, contribute to this process. Indeed, the pronounced growth of the stabilization energy is manifested in the row from chloroform to water (Table 1).

**Figure 4:**
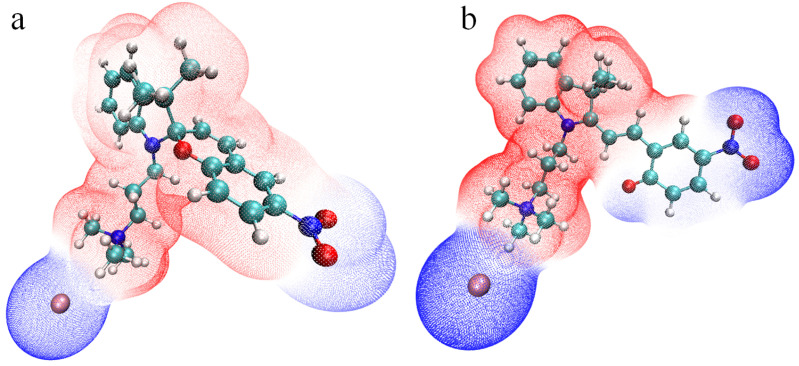
The surfaces of the electrostatic potential around spiropyran (**a**) and merocyanine (**b**) cations paired with bromide anion. The surface having a negative sign is colored in blue, the positive one-in red. This picture provides the differences in the *V*(**r**) caused by the isomerisation upon light. For this illustration, the following software is utilized: Multiwfn, Version 3.8 [77] and VMD, Version 1.9.3. [78]. The carbon atoms here are colored in cyan, the remaining atoms have colors as noted in Figure 3. The bond order is not shown.

The changes in the charges pattern are shown in Figure 4, which represents the electrostatic potential (ESP) around the closed (a) and opened (b) cations, including bromide anion next to the positively charged surfactant’s head. This property has been calculated for the optimized geometries in implicit water. The analysis of the ESP surfaces is a common approach to describe the ring-opening reaction of spiropyrans [75,76], to calculate the group charges accumulated on the acceptor and donor groups of the MC form as useful indicators to get insight into the charge separated state of the molecule [56,58], to shed light on the polarity of the protonated MC forms upon hydrolysis [59], for studying crystal packings of various spiropyrans [67]. The mentioned properties are assuredly important, but here we would like to emphasize that the charge redistribution appears also as a decisive factor of hydrophobicity change upon light stimulus, that has been anticipated in experiments [19,22,36].

Indeed, the closed SP form is less polar and consists of a chromene part with a strong electron-withdrawing nitro group, with ESP colored in blue together with bromide anion. The ring-opening isomerisation leads to the expansion of the negatively-charged region, which includes now phenolate anion almost completely (some atoms are carrying the partial charge close to zero). At the same time, the concentration of the positive charge is growing next to the nitrogen of the indoline moiety. Thus, the light stimulus converts the head-tail structure into a triangle-like shape with two positive-charged triangle vertices (quaternary ammonium headgroup and indoline nitrogen) and one negative-charged vertex (phenolate oxygen and nitro group). The redistribution of charges is reflected also in the static dipole moments of the isomers, cf. 21.8D and 28.7D for SP and MC, correspondingly.

The above-mentioned electrostatic potentials, the dipole moments, the experimental data on different behavior of the closed and opened forms measured in surface tension experiments [36] or in in-situ AFM studies [19] are all indirect evidences of more hydrophilic (solvophilic) nature of one isomer and more pronounced hydrophobicity (solvophobicity) of another one. The quantification of each isomer in this respect is possible in computer simulations applying the implicit solvation approach [79] and thermodynamic integration [3] with the aim to calculate the free energy of solvation (hydration).

**Figure 5:**
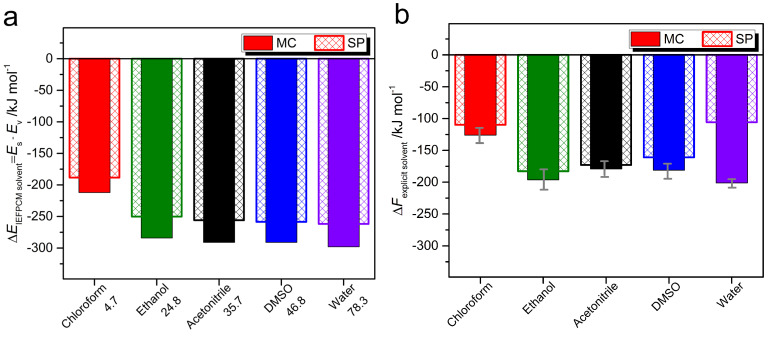
The energy difference Δ*E*${}_{IEFPCMsolvent}$ between the value of a cation in a particular solvent *E*${}_{s}$ and the same in vacuum *E*${}_{v}$, as calculated in DFT approach (**a**) and the Helmholtz solvation free energy Δ*F*${}_{explicit}$ ${}_{solvent}$ (**b**) for cations, as received in thermodynamic integration. The data for chloroform, ethanol, acetonitrile, dimethyl sulfoxide and water are shown in red, olive, black, blue and violet, respectively, for merocyanine (closed vertical columns) and spiropyran (shaded vertical columns). The numbers next to the solvents in panel (**a**) are the values of ϵ.

The results of both approaches are illustrated in Figure 5. The first obvious conclusion was that, independently on the solvent, the MC form is preferably solvated as compared to SP isomer. The tendency to preferential solvation is more pronounced for solvents with a higher polarity. The last observation follows from the Figure 5a, where the molecularity of a solvent is not included, since the surrounding medium is modeled as dielectric background. For the explicit solvation, when the intermolecular interactions with chloroform, ethanol, acetonitrile, dimethyl sulfoxide, and water are simulated in almost all their complexity, this trend still continues, though not as pronounced as in the previous case.

The largest difference in the Helmholtz free energy Δ*F*${}_{explicit}$ ${}_{solvent}$ for both isomers found for the hydration process (Figure 5b) is ca. 100 ± 4 kJ mol${}^{-1}$. This explains the isomerisation of SP into MC in aqueous media. When spiropyran-containing amphiphile is dissolved in water, an open-ring MC state is formed spontaneously through the cleavage of the C${}_{spiro}$–O bond due to high polarity of water. Only in this solvent among all simulated ones we could detect the hydrogen bonding between the phenolate oxygen and the water molecules with an average length of 1.94 ± 0.06 Å (between O${}^{-}$ and H${}_{water}$), which is strong, mostly covalent, according to the classification of Jeffrey [80].

The minor differences in the preferential solvation of MC over SP form, as found in TI, are obtained in other solvents with the values ranging from 5 to ca. 20 kJ mol${}^{-1}$. This means that one could not expect the spontaneous and detectable appearance of the merocyanine form upon dissolution of SP in organic solvents. In the next section we demonstrate the color changes upon dissolution, storage and light stimulus, and we have a chance to benchmark theoretical predictions and experimental findings.

### 2.2. Photophysical Properties

**Figure 6:**
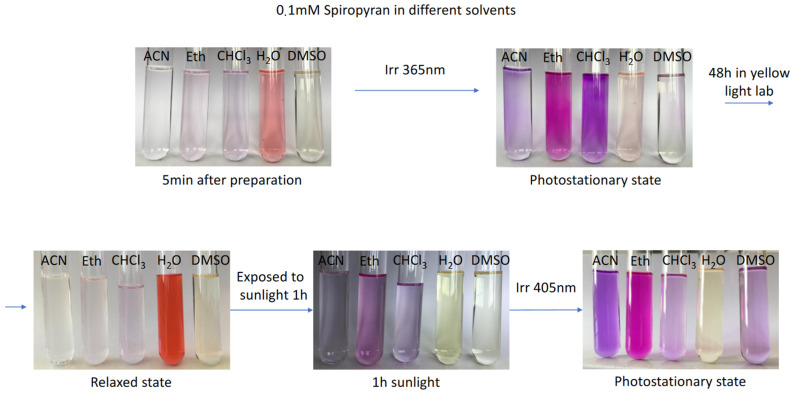
The “rainbow” of 0.1 mM spiropyran solution in different solvents under various light stimuli. The “yellow light” means light in the laboratory where the experiments are conducted, which is in our case 570 nm.

**Figure 7:**
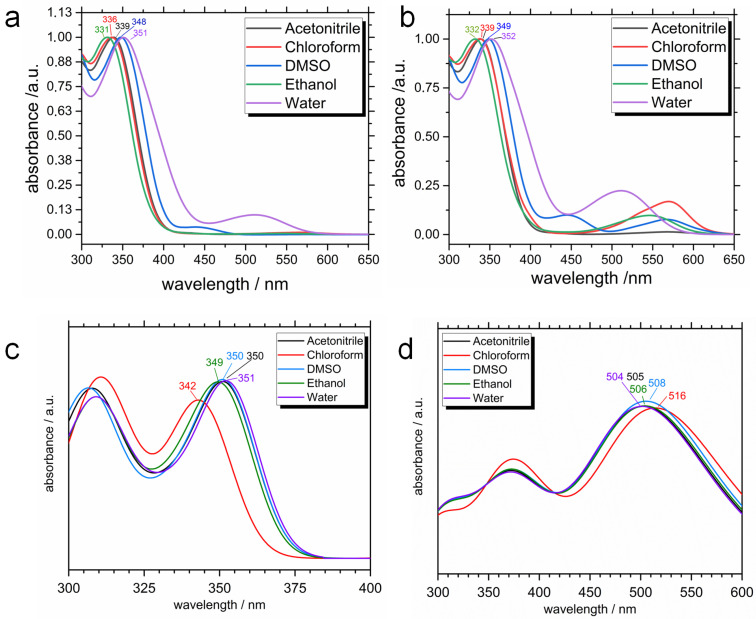
Normalized absorption spectra of 0.1 mM SP before irradiation: sample dissolved in appropriate solvent and measured after one hour standing in yellow light lab (**a**) and normalized absorption spectra of 0.1 mM SP after irradiation (**b**): samples irradiated with UV light until photostationary state is achieved. The calculated spectra (PBE/6-311G${}^{*}$) of SP (**c**) and MC (**d**) forms in different IEFPCM solvents. Computed spectra have been smoothed using Gaussian functions of a half-width at a half-height of 0.333 eV.

The most interesting phenomenon, which has been already mentioned, is a negative photochromism, which is illustrated in Figure 6 and Figure S1. Five minutes after dissolution is already enough to validate our theoretical results: initially colorless spiropyran turns orange in water, indicating the isomerisation to the colorful MC form. A characteristic peak is clearly seen around 510 nm before UV light irradiation in Figure 7a, which is a signature of the MC conjugated backbone. This peak is not present for all other solvents, and therefore for all other systems we have normal photochromic properties. Upon UV stimulus (Figure 7b), the MC absorption band appears for all the solvents. The bands are centered at a different wavelength from 510 to 570 nm, which could be explained by solvatochromism [81], specific solute-solvent interactions, and possibly by the formation of aggregates [35].

Under UV light stimulus (365 nm) at the photostationary state, the solutions are colored (Figure 6), showing a photoisomerization of SP to MC, i.e., positive photochromism for organic solvents. The aqueous system is slightly colored, which means that MC form is present in the solution, but its concentration is lower as compared to the just prepared mixture with water. Later, after 48 h in yellow light lab, the thermal back reaction happens, TMAB-C3-MC is converting to TMAB-C3-SP in organic solutions, but the relaxed state in water is deeply colored in orange-red color, showing larger MC content. Further, all the systems were exposed to sunlight for one hour, which promotes SP→MC transition, and solutions are colored again. Finally, the irradiation with λ = 405 nm enhances the solution’s colors.

Theoretical spectra calculated for the TMAB-C3-SP and TMAB-C3-MC cations with counterion in implicit solvents, shown in Figure 7c,d, demonstrate a fair agreement with the experiments. First of all, the absorption band for SP (Figure 7c), centered in experiments at 331–351 nm, appears in the same region in simulations (342–351 nm), and is blue-shifted for the solvents of lower polarity. The latter fact finds its corroboration in papers by Zhou et al. [68] and Barachevsky [69]. In TD-DFT (time-dependent DFT) calculations we always have one single isomer in an optimized geometry, and therefore, at this level of calculations we cannot see the isomerisation, and therefore, the appearance of new absorption bands. The calculated spectral characteristics for MC (Figure 7d) are characterized by the presence of two absorption bands, one in UV (at ca. 370 nm) and another one is in visible region (between 504 and 516 nm). Again, in experiments the absorption spectra of the isomer’s mixture are measured, and the absorption band in the UV region remains centered at the same wavelength as in spectrum of SP (cf. Figure 7a,b). In TD-DFT calculations, only MC isomer is considered, and only its excitations contribute to the spectrum. The most important conclusion here, is the absorption band in the visible region belongs to the same λ values, as obtained in experiments.

**Figure 8:**
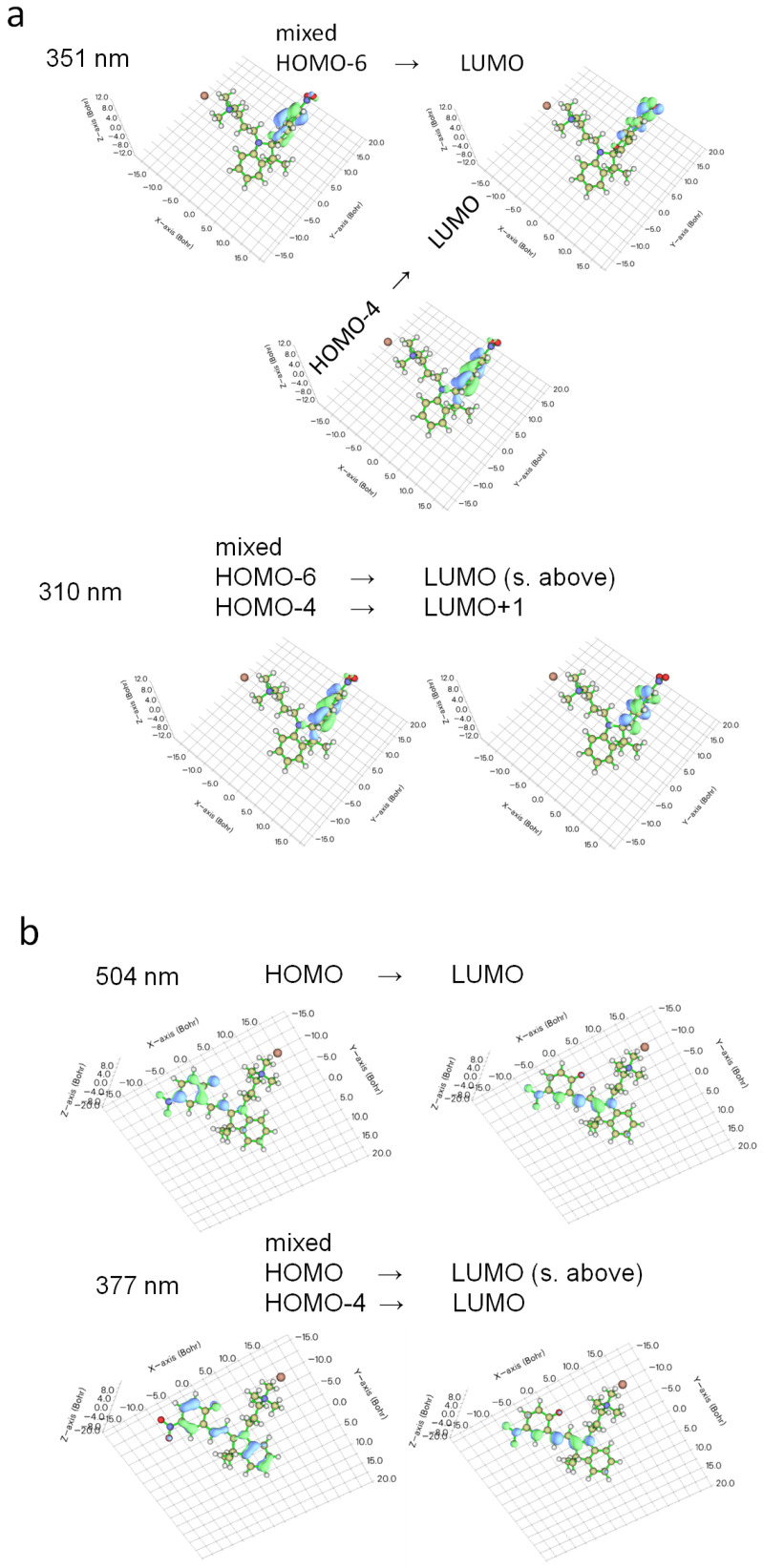
Molecular orbitals involved in the electronic transitions of different isomers at absorption bands shown in Figure 7c,d for SP (**a**) and MC (**b**). For this illustration, Multiwfn, Version 3.8 [77] has been used.

Some representative molecular orbitals involved in the electronic transitions are shown in Figure 8. For SP isomer in water (Figure 8a), the electronic transitions happen from HOMO-4 and HOMO-6 to LUMO at 351 nm and from the same occupied orbitals to LUMO and LUMO + 1 at 310 nm. These transitions reveal the localized nature of two molecular parts (indoline and chromene) separated by C${}^{spiro}$ atom. Similar pictures of the frontier orbitals, including those involved in the electronic transitions, have been found for spiropyran derivatives [35,56,82]. Here, the so-called intraligand excitations (IL) are observed: the intense absorption band at 342–351 nm (Figure 7c) corresponds to the $\pi -\pi^{*}$ transitions of the chromene moiety. In previous publications [35,83,84] the $\pi -\pi^{*}$ transition in chromene is also centered at ca. 340 nm. The band at shorter wavelengths (305–310 nm) happens from chromene and partially indoline to chromene. In the literature it has been reported that bands lying at shorter λ correlate to the $\pi -\pi^{*}$ transitions in indoline [35,85]. In some publications, for instance by Abdel-Mottaleb and Ali [75], the electronic transitions involved charge transfer from donor indoline to acceptor chromene (with nitro substituent) molecular fragment. Costa Duarte et al. [86] has reported that the excitation at $\lambda_{max}$ = 280 nm includes HOMO→LUMO $\pi -\pi^{*}$ transition, and both orbitals are delocalized over the whole molecule. The mentioned examples demonstrate that the molecular parts involved in a particular electronic transition are very much dependent on the substituents in the structure, which in turn influence the geometry, and therefore, the molecular aromaticity and conjugation.

Upon irradiation with UV light or after dissolution in water, the TMAB-C3-SP form is transformed into the opened TMAB-C3-MC form, accompanied by a solution color change. The visualization of the excitations in the MC isomer (Figure 8b) illustrates more delocalized conjugated nature of the molecule, which is supported by the majority of theoretical studies [56,71,75,87]. The excitations at 504–516 nm are attributed to the $\pi -\pi^{*}$ transition of the opened form [35].

In the last paragraph we would like to mention that calculated and measured photophysical spectra (mainly positions of the absorption bands and solvatochromism) are consistent with what is reported in the literature [18,19,22,24,37,45,56,58,62,75,81,87,88,89,90] findings for the positive photochromic spiropyran systems, as well as for still rare data concerning photoisomerization of SP/MC-containing surfactants, exhibiting negative photochromism.

### 2.3. Self-Assembly in Water

In order to simulate self-assembly, on one side we need to setup the system of relatively high molar concentration of the TMAB-C3-SP and TMAB-C3-MC, and on the other side, initially the molecules in the simulation box have to be surrounded by water molecules and do not have a direct solute–solute intermolecular contacts. For the simulations described in this section, the molar concentration is *C* = 0.36 mol L${}^{-1}$. This value may seem too high, and in the simulations of the photoresponsive amphiphiles, such as azobenzenes, the modeled values of *C* are usually lower (on the order of mmol L${}^{-1}$). However, the most important criterion is to set the concentration which is above CMC (critical micelle concentration). This condition is necessary and sufficient to test the stability of initially preorganized azobenzene micelles [91] or to see the self-assembling in solution [92].

**Figure 9:**
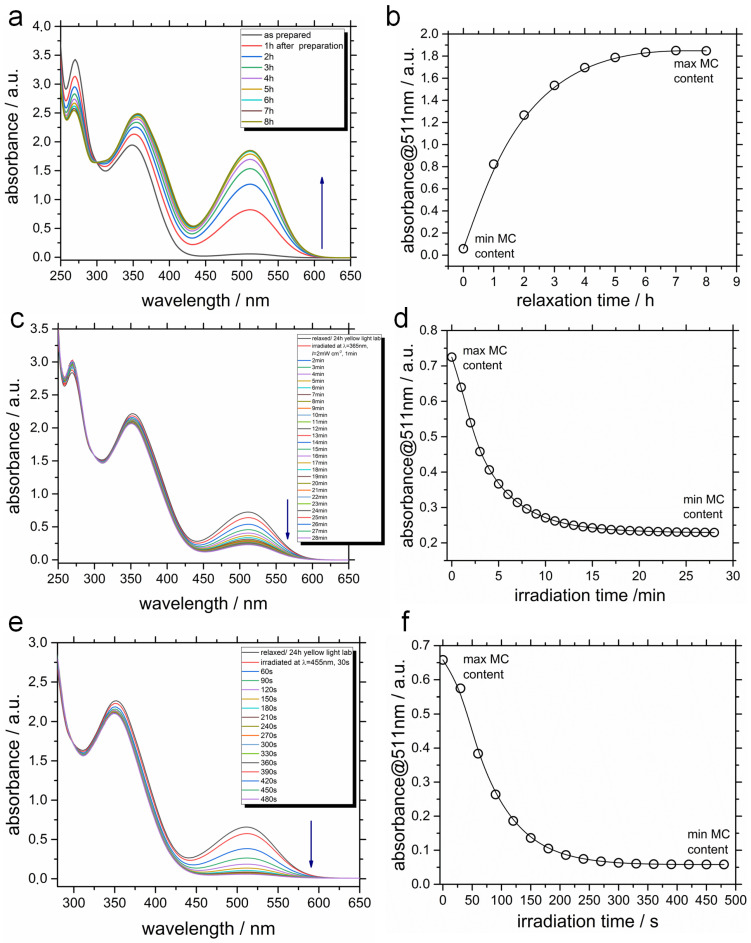
Normalized absorption spectra of 0.1 mM SP in water. (**a**,**b**)—Relaxation in the dark; a sample dissolved in water and placed directly in the spectrometer. (**c**,**d**)—The conversion kinetics of the relaxed state (24 h yellow light lab) to UV (365 nm) irradiated photostationary state; the irradiation intensity is 2 mW cm${}^{-2}$. (**e**,**f**)—The conversion kinetics of the relaxed state (24 h yellow light lab) to blue (455 nm) irradiated photostationary state; the irradiation intensity is 2 mW cm${}^{-2}$. The min MC and max MC content is 3 and 100% (panel b). A half-lifetime for complete switching to max MC containing photostationary state is 110 min. For panel d, the max MC is 39% and min MC is 12%. For panel f, these values correspond to 36 and 3%, respectively. These values are obtained using methodology described in our previous publication [93].

Let us finally consider the kinetics of the colorizing and photobleaching (Figure 9). This illustration has three rows of data: the panels a,b show the growth of the MC content starting from “as prepared” watery solution to the system after 8 h stored in the dark; the panels in the middle (c, d) provide us with the spectral characteristics of the MC solution initially relaxed for 24 h in the yellow light lab and then irradiated at λ = 365 nm during 28 min, and finally the row at the bottom (panels e, f) demonstrates the kinetics of MC→SP photoreaction under visible light at λ = 455 nm during 480 s.

Again, when surfactant TMAB-C3-SP is dissolved in water, the characteristic peak is clearly observed at 511 nm, and the solution color is orange. Interestingly, this peak completely disappears under visible light accompanied by photobleaching during next 8 min (Figure 9e,f). Similar behavior is observed in papers by Sakai et al. [18], Zhou et al. [68], Han et al. [24]. One more fact deserving our close attention is that UV light, which is supposed to promote the photoisomerization from SP into MC, acts in a way of decreasing the MC content, bringing this value to that one corresponding to the concentration of MC at the photostationary state (Figure 9c,d). This is true, however, for a solution which is stored for 24 h in the dark, where the isomerisation happens spontaneously in a polar aqueous environment. We should also note here that hydrolysis or protonation of TMAB-C3-MC does not occur, for that the acidic or alkaline environment is required [37]. All the measurements are conducted at pH 7.

Another important note here is that we put an emphasis on the evaluation of the molecular binding in the aggregate, rather than on the micellar shape, which depends on the solution concentration both for conventional and photoresponsive surfactants [3]. However, it is still interesting to know *a priori* which aggregate shape is encoded in the molecular geometry, as it has been realized by us for azobenzene amphiphiles [3]. To do this, the Israelachvili’s critical packing parameter *P* [94] has been calculated for SP and MC isomers. Not surprisingly, in view of the quite voluminous surfactant tail, the *p* values are high and very close to each other: *P*${}_{MC}$ = 0.97 and *P*${}_{SP}$ = 0.95, which indicates that both isomers are prone to form lamellar structures or bilayer vesicles—some elongated, non-spherical shapes.

**Figure 10:**
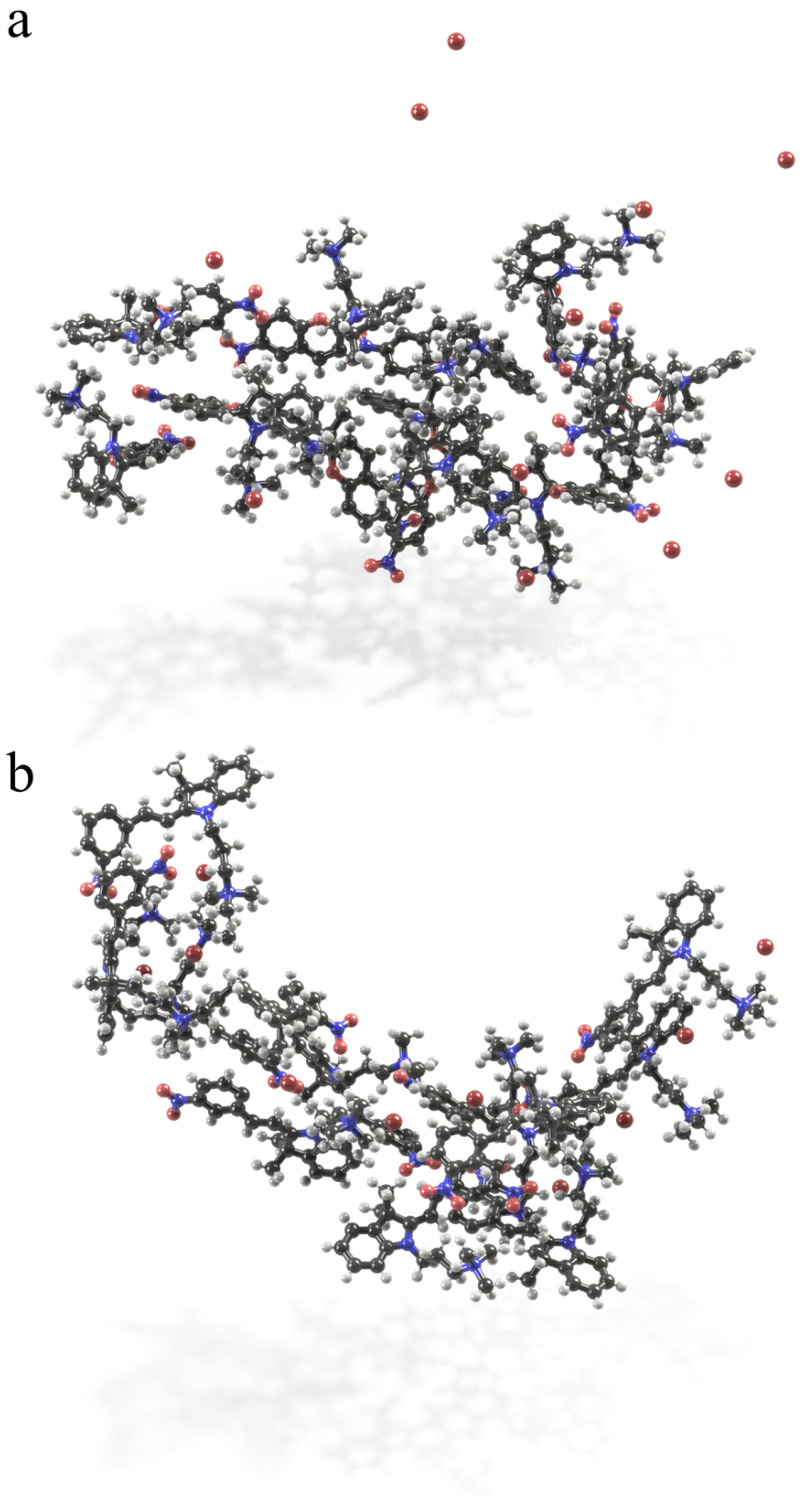
The self-assembled structures of the SP (**a**) and MC (**b**) as found in MD simulations in water. The aggregates consist of 12 molecules and 12 counterions, which in the case of SP are only partially condensed on the micelle. Less elongated aggregate is observed for spiropyran with the Connolly surface occupied volume of 5201.3 Å${}^{3}$ and the surface area of 4238.0 Å${}^{2}$. More extended micelle is built from MC form. Here, the Connolly surface occupied a volume of 5621.3 Å${}^{3}$ and a surface area of 4066.4 Å${}^{2}$. For MC, the Br${}^{-}$ ions are predominantly in the vicinity of the TMAB head.

**Figure 11:**
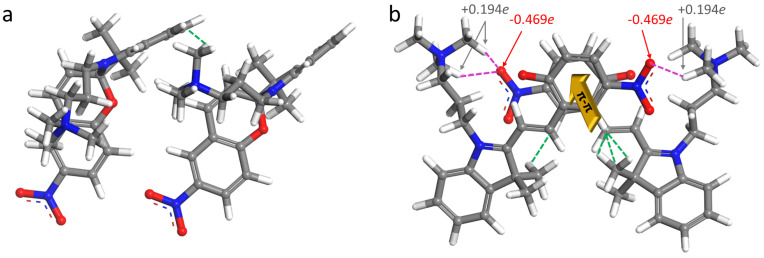
The snapshots of the pairs of SP (**a**) and MC (**b**) after geometry optimization. The structure (although we cannot call it the prevailing one, since SP dimers do not have a pattern in common) of the SP dimer given as an example (**a**). This dimer is stabilized apparently by van der Waals intermolecular contacts (green dashed line). The MC dimer (**b**) depicted here has a typical mutual arrangement of the molecules. The interactions stabilizing this micellar building block are the π−π interaction schematically shown by a yellow arrow, the van der Waals contacts of alkyls and aryls (green dashed lines), and the electrostatic attractions (crimson dashed lines) related to the pairing of partially negatively charged oxygens of nitro groups with positively charged hydrogens of trimethyl ammonium of a neighboring molecule. Both van der Waals and electrostatic through-space couplings happen on both sides of the stacked dimer. An average length of the π−π distance is 4.11 ± 0.51 Å, of the electrostatic interactions is 2.19 ± 0.07 Å, and the van der Waals contacts are 2.40 ± 0.19 Å in length. Partial charges of O(–NO${}_{2}$) and H(–N(CH${}_{3}$)${}_{3}$) are written in red and gray color, respectively. Visualization is done in Materials Studio 9.0 [96].

The snapshots of self-assemblies in solutions are shown in Figure 10. Non-planar, less hydrophilic molecules of spiropyran in the aggregate are disarranged, however, the charged TMAB heads are pointing toward water. This aggregate is less elongated as compared to the MC micelle. The latter one is more extended. This conclusion follows from the comparison of the Connolly surface occupied volumes and areas (Figure 10). Being flat and conjugated, merocyanine forms stack-like structures (Figure 11), in which two-three molecules are bound through π−π interaction, similarly to the results of Zhang et al. [35,95]. For MC micelle, almost all three charged vertices are exposed to water, and the stacks are formed in a way of maximizing the interactions between conjugated backbones.

Interestingly, the π−π interactions gather the MC molecules in stacks, and these stacks are additionally stabilized by van der Waals contacts and electrostatic attractions (Figure 11). Hence, the van der Waals coupling of alkyls and aryls is characterized by the distances of ca. 2.40 ± 0.19 Å between the atoms in a contact. The electrostatic attractions related to the pairing of partially negatively charged oxygens of nitro groups O(–NO${}_{2}$) with positively charged hydrogens of trimethyl ammonium H(–N(CH${}_{3}$)${}_{3}$) lead to shorter intermolecular contacts of 2.19 ± 0.07 Å. Such a stack represents a repeating pattern in the MC aggregate. We could imagine that this can be considered as a building block of some cylinders with stacks in the middle and charged and hydrophilic exterior shell. The SP aggregate does not have any order in the structure, any common building block, and the interactions which held the molecules together are van der Waals ones. SP molecules cannot approach each other closer because of both non-planarity of the molecule and the presence of the side chains. However, these molecules could build self-assembled monolayers at the interface owing their amphiphilic nature.

Finally, in order to quantify the intermolecular interactions, the binding energy in dimers *E*${}_{b}$ is calculated. A hundred of snapshots is taken from the MD trajectories for both isomers. Further, the interacting cations without Br${}^{-}$ counterions are cut from each frame (up to six pairs) and their geometry is optimized in pairs. The optimization settings are the same, as for the isolated molecules. The energy of each molecule in dimer is found in a single-point DFT calculation. Here, the molecules have geometries as they adopt in the interacting dimer after the optimization procedure. The *E*${}_{b}$ is the difference between the energy of dimer and the sum of energies of each molecule in this couple [3]:

(1) $$E_{b}=E_{dimer}-\left(E_{1}+E_{2}\right)$$

Using this equation, the following energies are obtained: The binding of MC molecules proceeds very efficiently with *E*${}_{b}$ ranging from −53.4 to −11.9 kJ mol${}^{-1}$. For SP dimers, the interacting energies are not always stabilizing ones, showing their range from negative (−6.4 kJ mol${}^{-1}$) to positive (+17.6 kJ mol${}^{-1}$) values, depending on mutual orientations of the molecules. It would be very interesting to evaluate the binding energies, for example in MC stacks studied in NCI calculations by Zhang et al. [35,95] and compare them with coupling between the non-stacked SP molecules in pairs taken from experimentally resolved crystal unit cells (e.g., CCDC 2015826) [35]. This task as well as theoretical analysis and systematization of published experimental data could be a topic for future research.

## 3. Discussion

In this joint experimental-theoretical work, we have investigated various properties of the amphiphilic molecule with trimethylammonium charged head and spiropyran-containing tail, which are interconnected by a relatively short propyl hydrocarbon chain. Being dissolved in tested organic solvents, the system demonstrates positive photochromism, i.e., upon UV stimulus the colorless spiropyran form is transformed into colorful merocyanine isomer. However, the aqueous solution of the amphiphile possesses a negative photochromism: the orange-red merocyanine form becomes thermodynamically more stable in water, than its closed isomer, and both UV and vis stimuli lead to the partial or complete photobleaching of the solution, respectively. The explanation of this phenomenon is given on the basis of DFT calculations and classical modeling including thermodynamic integration. The simulations reveal that stabilization of MC in water proceeds with the energy of ca. 70 kJ mol${}^{-1}$, and that the Helmholtz free energy of hydration of this form is 100 kJ mol${}^{-1}$ lower as compared to the behavior of SP isomer in water. The explanation of such a difference lies in the molecular properties of the MC: after the ring-opening reaction this molecule transforms into a zwitterionic form, as evidenced by the electrostatic potential plotted around the opened form. The presence of three charged groups on the periphery of a flat conjugated backbone stimulates the self-assembly of MC molecules in water, and not just gathering of the molecules into some aggregate without a molecular order in it, as in case of SP form, but in some elongated associates with stack-like building blocks, as shown in MD simulations of the aqueous solution with the concentration above CMC.

Our quantitative evaluation of the hydrophilicity switching in SP/MC containing surfactants may prompt the search for new systems, including colloidal and polymeric ones, aiming at remote tuning of their morphology, which in turn means control over a more complicated material’s applications. One recent example is described in a paper by Kim et al. [22]. We also believe that the effect, which is less strong in organic media, offers more gentle levers, shows subtle effect on morphology restructuring, which could give new promising shapes and patterns.

A drastic hydrophilicity change occurring in water could also be of importance for experiments related to biology and medicine, for example, for cell manipulations, since spiropyran-based surfactants showed negligible toxic effects on the cells up to millimolar concentrations [97], as compared to azobenzene-based soaps. Studied amphiphiles could be implemented in biomolecular experiments, where researchers need a quick photoresponse, or changing amphiphilic balance with time just dissolving TMAB-C3-SP in water, e.g., for further development of drug delivery systems.

## 4. Methods and Materials

### 4.1. Materials

**Figure 12:**
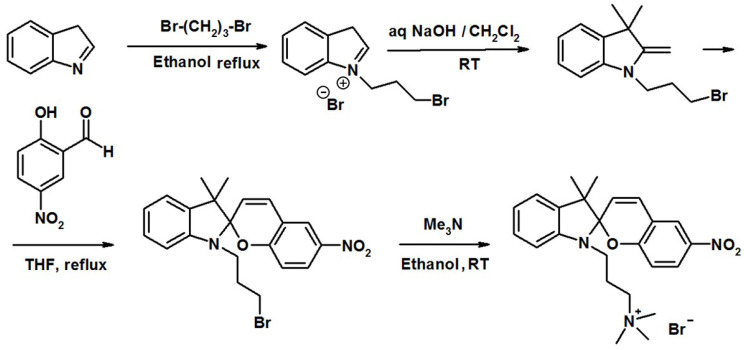
The scheme of synthesis of the 1${}^{\prime }$-(3${}^{\prime \prime }$-trimethylammoniopropyl)-3${}^{\prime }$,3${}^{\prime }$-dimethyl-6-nitrospyro(2H-1-benzopyran-2,2${}^{\prime }$-indoline) bromide according to Hammarson et al. [11].

1${}^{\prime }$-(3${}^{\prime \prime }$-trimethylammoniopropyl)-3${}^{\prime }$,3${}^{\prime }$-dimethyl-6-nitrospyro(2H-1-benzopyran-2,2${}^{\prime }$- indoline) bromide is synthesized as described elsewhere (Figure 12) [11]. The product is a spiropyran containing cationic surfactant with a spacer of three methylene groups between the positively charged trimethylammonium bromide (TMAB) head group and the photoresponsive unit.

### 4.2. Methods

#### 4.2.1. Time Resolved UV-Vis Measurements

Time resolved UV-Vis measurements are performed with a commercial Cary 5000 UV-Vis-NIR spectrophotometer instrument (Agilent Technologies, Santa Clara, CA, USA). A rectangle quartz cuvette transparent in all directions (Hellma Analytics, Müllheim, Germany) of 1 cm thickness is used for the measurements, the surfactant concentration is 0.1 mM. Irradiation of the spiropyran solutions is done by two light sources UV (365 nm) and blue (455 nm) LED lamps (Thorlabs, Newton, MA, USA) directly in spectrometer until reaching the photostationary state of the solution. The intensity of light for both wavelengths is 2 mW/cm${}^{2}$ measured with a commercial S170C power meter (Thorlabs, Newton, MA, USA).

#### 4.2.2. DFT and Time-Dependent DFT Calculations

All DFT calculations are carried out with Gaussian 09 Revision C.01 suite [74]. The geometry optimization of SP and MC is performed in a gas phase using the B3LYP/DGDZVP basis set with a tight self-consistent field convergence threshold. This functional is widely and successfully employed in studies of spiropyran-containing molecules [10,11,49,57,58,59,60]. Next, the ground state geometries are optimized by applying the integral equation formalism variant of the polarizable continuum model to simulate the solvent environment [72]. In this model, the molecular cavity is constructed applying the Universal Force Field [73]; the shape of the cavity is defined by interlocking spheres centered on each solute atom having van der Waals radii scaled by a factor 1.1. Harmonic vibrational frequency calculations are used to confirm that the structure of each isomer in vacuum and in IEFPCM/UFF solvent is a true minimum, to obtain the standard enthalpy of formation and to calculate the zero-point energy correction at 298 K. The solvents mimicked in the DFT calculations are: chloroform (ϵ = 4.7113 ), ethanol (ϵ = 24.852), acetonitrile (ϵ = 35.688), dimethyl sulfoxide (ϵ = 46.826), and water (ϵ = 78.3553).

The vertical transition energies to the first 100 excited states are calculated for each isomer having optimized geometries in a particular solvent using time-dependent DFT with the B3LYP [58], PBE [87] functionals and the DGDZVP and 6-311G${}^{*}$ basis set, correspondingly. The DGDZVP basis set is shown to predict the energies close with respect to the experiment transition energies, e.g., for merocyanines [88]. The combination B3LYP/6-311G${}^{*}$ has also shown a good agreement with measured spectra [75]. The electronic spectra are simulated employing Gaussian functions with the half-width at half height of 0.333 eV to build a continuous spectrum from a collection of transition peaks corresponding to the time-dependent DFT transition energies and oscillator strengths. For the qualitative description of the electronic transitions, the natural transition orbitals [98] for the excited state of interest are analyzed.

The solvent-accessible surface area of the TMAB head (*a*) and the occupied molecular volumes (*v*) are calculated for the optimized geometries in implicit water [96]. The rolling probe radius is 1.4 Å for water [99]. These quantities, as well as the length of the hydrophobic tail (*l*) of the amphiphile are further used for the calculation of the Israelachvili’s critical packing parameter *P* [94], as described in our previous publications [3,100]. The *p* value predicts possible micellar structures that could be built by a surfactant in water (Equation (2)):

(2) $$P=\frac{v}{a\times l}$$

The electrostatic potential *V*(**r**) is calculated using the Merz-Singh-Kollman procedure [101] for the molecular cations and for the cations paired with bromine anion and mapped onto a surface with an electron density isovalue of 0.02 au. The ESP (Equation (3)) is defined as the interaction energy between the electrical charge generated from the molecule electrons and a positive point charge as a probe located at the position **r**:

(3) $$V\left(r\right)=\sum_{A=1}^{N}\frac{z_{A}}{|R_{A}-r|}-\int \frac{\rho \left(r^{\prime }\right)d^{3}r^{\prime }}{|r-r^{\prime }|}$$

Here *z${}_{A}$* is the charge of the nucleus *A*, which is a point charge located at *R*${}_{A}$. The term $\rho (r^{\prime }$) is the electron density function. The sign of the electrostatic potential is correlated to the partial charges on the atoms/atomic groups, i.e., the value at the minimum of V(r) quantifies the electron-rich character of that region, and vice versa. The initial guess for the Br${}^{-}$ anion placement next to the molecular cation is made using the analysis of the partial ESP charges.

#### 4.2.3. Thermodynamic Integration

Prior to use in thermodynamic integration, the starting configurations of the cubic simulation boxes have been prepared following the scenario below. First, ten systems are constructed for five solvents and two cationic isomers paired with Br${}^{-}$. Each simulation box contained 500 solvent molecules, one cation and one anion. Initially, the systems are composed with reduced densities $\rho_{i}$, as given in Table 2. This step is undertaken to avoid both close atom contacts and steric intermolecular clashes.

Next, classical 2ns NPT molecular dynamics simulations at *T* = 25 ${}^{\circ }$C and *P* = 10${}^{-4}$ GPa controlled by a Nosè thermostat with the *Q* ratio of 0.01 and Berendsen barostat with decay constant of 0.1 ps, respectively, are performed using the universal force field (UFF) [73] as implemented in Materials Studio 9.0 [96]. The UFF has been applied in the majority of the simulation works related to spiropyran/merocyanine pair in various solvents [58,103]. During this MD phase, the systems reached $\rho_{r}$ densities, that are close to the experimental values ρ of pure solvents [102]. At this stage and in the following, water molecules are modeled using UFF with TIP3P charges (oxygen −0.834*e* and hydrogen +0.417*e*). The ESP charges on atoms of acetonitrile, ethanol, dimethyl sulfoxide and chloroform obtained in DFT calculations, are used as partial charges in MD runs. To account for the van der Waals interactions, we use the atom-based summation method and the cubic spline truncation approach with a cutoff at 12.5 Å. For the calculation of the electrostatic interactions, the Ewald sum is applied. The partial charges on SP and MC atoms are the ESP ones, the Br counterions are carrying the charge −1.

The changes in Helmholtz solvation free energy Δ*F*${}_{explicit}$ ${}_{solvent}$ of the cationic SP and MC surfactants [3], i.e., the energy to transfer the amphiphile from its vapor phase (vacuum) to a solvent, is obtained by using a thermodynamic integration scheme implemented in Materials Studio 9.0 [96]. In this method, the solvation free energy is a sum of three contributions: the ideal, the van der Waals, and the electrostatic one. The first term is the ideal contribution. Starting from a surfactant molecule in vacuum, the ESP atomic partial charges are gradually reduced to zero, while keeping all other interactions the same. The second one, called the van der Waals term, is calculated when the non-charged amphiphile is coupled to the solvent by switching on the van der Waals interactions. Finally, the electrostatic contribution, i.e., the free energy of charging the cavity once it has been placed into the solution, is defined. The calculation of each contribution consists of a series of molecular dynamics calculations in NVT (canonical) ensemble, in which the interaction strength is effectively modified through the coupling parameter λ. The λ value defines the strength of the interaction between the solute and the solvent being represented by a number between 0 and 1, increasing its value by 0.01 for each run (λ = 0 means no coupling while λ = 1 denotes a fully coupled solute and solvent). Once the systems consisting of a solvent (500 molecules), surfactant in the SP or MC form and Br${}^{-}$ ion are pre-equilibrated (0.1 ns NVT run), the simulation is followed by a new equilibration for 0.5 ns and a productive NVT MD simulation for 1ns at each λ value. The Δ*F*${}_{explicit}$ ${}_{solvent}$ is calculated in each molecular dynamics run. At least three independent calculations of the Helmholtz free energy of solvation are performed for SP and MC form. More details on the Δ*F*${}_{explicit}$ ${}_{solvent}$ calculations can be found in a paper by Montagna et al. [3].

#### 4.2.4. Molecular Dynamics Simulation of Self-Assembly in Aqueous Phases

The MD simulations of the SP or MC assembly in explicit water are conducted in a cubic box containing 12 cationic surfactants of each type, 12 Br${}^{-}$ ions and 1500 water molecules at initial density of 0.800 g cm${}^{-3}$. The equilibration of the systems is carried out in NPT MD simulation as described above. During this step, the solution density reaches the normal value of 0.960 g cm${}^{-3}$ (the density of organic systems in water at ambient conditions, the edge length of the simulation box is 37.7 Å). This number of solute molecules in a simulation box with water corresponds to the molar concentration of 0.36 mol L${}^{-1}$. Starting from these configurations, the simulations in the NVT ensemble for 12 ns are conducted at *T* = 25 ${}^{\circ }$C. The integration step in MD is 1 fs, the coordinates of all atoms are saved every 5 ps. The energy and temperature are monitored to conclude about the equilibration of the systems. All other MD settings are as above-described.

## Figures and Tables

**Table 1:** The energy difference Δ*E* (kJ mol${}^{-1}$) between the total energy of the MC cationic form (*E*${}_{MC}$) taken as a reference and the total energy of the SP cationic form (*E*${}_{SP}$). The total energies of the isomers are obtained in DFT calculations in the IEFPCM (integral equation formalism variant of the polarizable continuum model [72]) solvents with the cavity of the solute built up by using atomic radii from the universal force field (UFF) [73] by placing individual spheres around each heavy and hydrogen atom. The values of dielectric permittivity are used in the calculations [74].

Solvent	Dielectric Permittivity ϵ	MC Form, Δ*E*	SP Form, Δ*E* = *E*${}_{\mathrm{MC}}$ – *E*${}_{\mathrm{SP}}$
chloroform	4.7113	0	+58.02
ethanol	24.852	0	+67.52
acetonitrile	35.688	0	+68.77
dimethyl sulfoxide	46.826	0	+68.76
water	78.3553	0	+70.21

**Table 2:** Solvents simulated in this work, their densities ρ at 25 ${}^{\circ }$C [102], initial values $\rho_{i}$ of the solutions for the construction of the simulation cells (so called diluted) and resulting densities $\rho_{r}$ of the solutions after a 2 ns NPT MD run (isothermal-isobaric ensemble) at *T* = 25 ${}^{\circ }$C and *P* = 10${}^{-4}$ GPa.

Solvent	ρ (g cm${}^{-3}$) at 25 ${}^{\circ }$C [102]	$\rho_{i}$ (g cm${}^{-3}$)	$\rho_{r}$ (g cm${}^{-3}$)
chloroform	1.4830	1.200	1.321(MC) 1.340(SP)
ethanol	0.7848	0.500	0.743(MC) 0.742(SP)
acetonitrile	0.7860	0.500	0.741(MC) 0.743(SP)
dimethyl sulfoxide	1.0958	0.800	1.032(MC) 1.042(SP)
water	0.9974	0.800	0.980(MC) 0.982(SP)

## Data Availability

Data is contained within the article or supplementary material.

## Supplementary Materials

The following supporting information can be downloaded at: https://www.mdpi.com/article/10.3390/ijms231911535/s1.

## Abbreviations

The following abbreviations are used in this manuscript:

SP	Spiropyran
MC	Merocyanine
DFT	Density functional theory
MD	Molecular dynamics
TI	Thermodynamic integration
