# Supplementary material for: Spiropyran/Merocyanine Amphiphile in Various Solvents: A Joint Experimental–Theoretical Approach to Photophysical Properties and Self-Assembly
Source: Int J Mol Sci. 2022 Sep 29;23(19):11535. doi: 10.3390/ijms231911535 (PMC9569490; doi:10.3390/ijms231911535)
Supplement: Supplementary file 1 [file ijms-23-11535-s001.zip › ijms-1906462-supplementary.pdf]

**Additional experimental data; Cartesian coordinates of SP and MC isomers in their optimized geometries in simulated implicit solvents.**

**Figure S1.** The color changes of 0.1 mM spiropyran aqueous solution under various light stimuli.

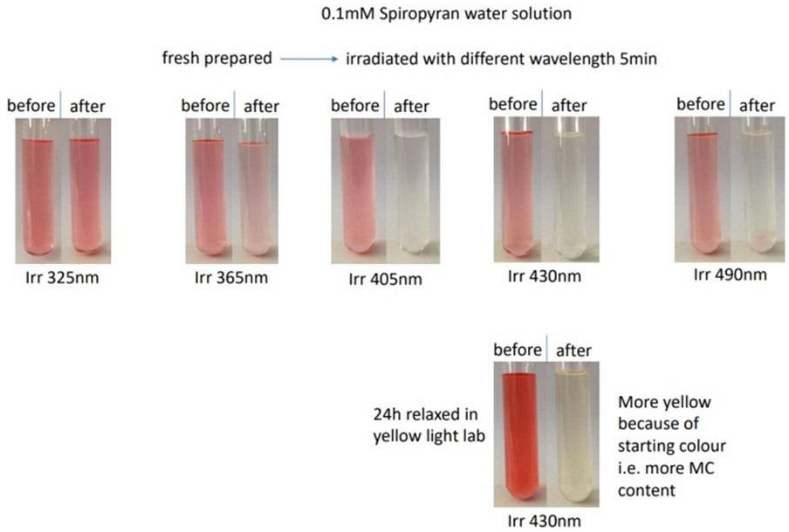


We would like to have a closer look at the reversible changes in sample color in aqueous solution (Figure S1). All fresh prepared solutions have orange color. Under UV light irradiation (at λ=325 and 365nm), the color remains orange which signals about the MC content in the sample, but partial color loss occurs already at 365nm. The visible light incident on the merocyanine system leads to photobleaching of the solution (irradiation at λ =405, 430, and 490nm), i.e. to MC->SP transition. The most dramatic change in the solution color, the clearest confirmation of the negative photochromism is seen in the last picture of Figure S1, where a deep orange-red MC system is converting to the original SP achromatic solution.

**MC (water)**

| | | | |
|---|---|---|---|
| Br | 6.41806700 | -2.30553500 | -0.59001200 |
| C | -0.13002400 | 6.12446400 | 0.12851200 |
| C | 0.01141700 | 4.84247900 | 0.67826900 |
| C | -1.00833100 | 3.92634500 | 0.42230900 |
| C | -2.12994700 | 4.24240700 | -0.34753000 |
| C | -2.26189300 | 5.51753500 | -0.88716800 |
| C | -1.25069200 | 6.46102600 | -0.64342900 |
| C | -3.03901300 | 3.02847200 | -0.43537500 |
| C | -2.24716800 | 1.99846500 | 0.38813600 |
| N | -1.12186100 | 2.57894600 | 0.85585900 |
| C | -3.20145500 | 2.58759700 | -1.91291100 |
| C | -4.41159100 | 3.34161500 | 0.21706100 |
| C | -0.10050700 | 1.93492300 | 1.69038600 |
| C | 0.97082600 | 1.22465400 | 0.84016600 |
| C | 2.02276700 | 0.61852600 | 1.77020400 |
| N | 3.15610400 | -0.13406600 | 1.08367700 |
| C | 4.09422100 | -0.63529100 | 2.15972300 |
| C | 3.93576500 | 0.76551900 | 0.15363100 |
| C | 2.64125300 | -1.32318200 | 0.30732900 |
| O | -2.10137400 | -1.98655300 | 1.77955200 |
| C | -3.20767200 | -2.31054900 | 1.27336600 |
| C | -4.04534800 | -1.37451800 | 0.49065800 |
| C | -3.69069100 | -0.01462500 | 0.24048700 |
| C | -2.55190400 | 0.64829700 | 0.66609600 |
| C | -5.26782000 | -1.82641400 | -0.04867500 |
| C | -5.70726500 | -3.13044500 | 0.14049300 |
| C | -4.92620600 | -4.05777100 | 0.89632400 |
| C | -3.73521500 | -3.65963900 | 1.43443300 |
| N | -6.95298100 | -3.54778800 | -0.42597300 |
| O | -7.62894200 | -2.73066600 | -1.09006700 |
| O | -7.33111200 | -4.72593900 | -0.23894900 |
| H | 0.64485600 | 6.86489000 | 0.30600400 |
| H | 0.88575000 | 4.59524700 | 1.27131800 |
| H | -3.12692800 | 5.78754800 | -1.48727600 |
| H | -1.33699600 | 7.46148300 | -1.05801700 |
| H | -3.64662100 | 3.40730300 | -2.48364800 |
| H | -3.85409800 | 1.71760700 | -2.00989800 |
| H | -2.23359900 | 2.34582800 | -2.36114500 |
| H | -4.29185800 | 3.64022400 | 1.26250600 |
| H | -5.08602900 | 2.48370000 | 0.17876800 |
| H | -4.88485800 | 4.16676400 | -0.32256900 |
| H | -0.59254000 | 1.23721400 | 2.36887700 |
| H | 0.34912800 | 2.71043900 | 2.31260900 |
| H | 1.42029000 | 1.95193300 | 0.16030900 |
| H | 0.48574400 | 0.45459400 | 0.23609500 |
| H | 1.56308400 | -0.09550900 | 2.45709400 |
| H | 2.50099000 | 1.39960100 | 2.36523100 |
| H | 4.90278600 | -1.18543700 | 1.67654400 |
| H | 3.53886700 | -1.28739600 | 2.83362500 |
| H | 4.48881500 | 0.22032800 | 2.70753300 |
| H | 3.30526800 | 1.05676500 | -0.68450900 |
| H | 4.79684800 | 0.20334700 | -0.21015800 |
| H | 4.26021000 | 1.64747500 | 0.70618100 |
| H | 2.05809200 | -1.95604000 | 0.97652500 |
| H | 3.50367100 | -1.86830200 | -0.07956600 |
| H | 2.02170700 | -0.97709000 | -0.51765000 |
| H | -4.41758400 | 0.53240300 | -0.35163900 |
| H | -1.85129900 | 0.06889200 | 1.25193300 |
| H | -5.88488300 | -1.14456100 | -0.62526600 |
| H | -5.29218000 | -5.06975400 | 1.03197300 |
| H | -3.12954600 | -4.35391100 | 2.01091700 |

**SP (water)**

| | | | |
|---|---|---|---|
| C | -5.86456900 | -3.15967800 | 0.03400700 |
| C | -4.50634000 | -2.84536900 | -0.02757700 |
| C | -4.13035700 | -1.51734700 | 0.12491300 |
| C | -5.09271200 | -0.52398700 | 0.31667800 |
| C | -6.43226600 | -0.84457500 | 0.37805700 |
| C | -6.82417000 | -2.17756800 | 0.23288000 |
| C | -4.40071100 | 0.80656900 | 0.50291200 |
| C | -2.98180900 | 0.47968000 | -0.10305200 |
| N | -2.84037300 | -0.96504500 | 0.12333700 |
| C | -4.28807700 | 1.09880000 | 2.00771800 |
| C | -5.09230300 | 1.97898600 | -0.18708800 |
| C | -1.76108200 | -1.71481600 | -0.50554400 |
| C | -0.36683600 | -1.25146700 | -0.08682700 |
| C | 0.65016100 | -2.27773200 | -0.55782800 |
| N | 2.09787900 | -1.89984600 | -0.34529600 |
| C | 2.94568000 | -3.06054300 | -0.76417600 |
| C | 2.37638500 | -1.60245100 | 1.09355700 |
| C | 2.48091200 | -0.71580000 | -1.17568900 |
| O | -1.97220300 | 1.14816500 | 0.67520600 |
| C | -1.13651300 | 2.07671900 | 0.17853400 |
| C | -1.09972700 | 2.39408100 | -1.18597400 |
| C | -2.01006700 | 1.67863500 | -2.06712300 |
| C | -2.87579900 | 0.80501700 | -1.56661700 |
| C | -0.19685500 | 3.34850500 | -1.62724000 |
| C | 0.64534200 | 3.96481400 | -0.71503100 |
| C | 0.61624500 | 3.64789600 | 0.63875100 |
| C | -0.27794500 | 2.69684200 | 1.08425500 |
| N | 1.58871600 | 4.96571500 | -1.18896600 |
| O | 1.60007800 | 5.23456500 | -2.38004300 |
| O | 2.32974600 | 5.49478300 | -0.37538700 |
| H | -6.16951500 | -4.19372200 | -0.08498900 |
| H | -3.78168300 | -3.63232700 | -0.19381000 |
| H | -7.17850900 | -0.07170900 | 0.53072700 |
| H | -7.87475800 | -2.44186300 | 0.26859900 |
| H | -3.75480700 | 0.30698300 | 2.53496200 |
| H | -5.29213400 | 1.17249800 | 2.42979900 |
| H | -3.77071100 | 2.04075200 | 2.19244000 |
| H | -5.28992100 | 1.78654000 | -1.24161800 |
| H | -4.48962100 | 2.88727400 | -0.11315100 |
| H | -6.04827300 | 2.18347700 | 0.29893400 |
| H | -1.84711300 | -1.69947500 | -1.59992800 |
| H | -1.88316500 | -2.75253500 | -0.19535900 |
| H | -0.34304700 | -1.14561500 | 0.99771900 |
| H | -0.15029100 | -0.27720900 | -0.52171700 |
| H | 0.54655800 | -2.46800700 | -1.62628400 |
| H | 0.50728300 | -3.22341000 | -0.03554600 |
| H | 3.99009100 | -2.78710800 | -0.63186900 |
| H | 2.74207000 | -3.28550500 | -1.80811600 |
| H | 2.69903000 | -3.91842000 | -0.14389500 |
| H | 1.83179800 | -0.71153400 | 1.39038300 |
| H | 3.44583800 | -1.43484000 | 1.20065800 |
| H | 2.06280600 | -2.45187000 | 1.69575300 |
| H | 2.26809500 | -0.93475500 | -2.21925600 |
| H | 3.54495500 | -0.53784200 | -1.03543200 |
| H | 1.91776600 | 0.15410100 | -0.85292400 |
| H | -1.96541300 | 1.87371700 | -3.13292100 |
| H | -3.55918800 | 0.26318200 | -2.21059300 |
| H | -0.14656000 | 3.60906700 | -2.67594400 |
| H | 1.28608000 | 4.14284900 | 1.32752200 |
| H | -0.32735000 | 2.42454200 | 2.13083200 |
| Br | 6.18689300 | -0.99409500 | 0.24687500 |

**MC (ethanol)**

| | | | |
|---|---|---|---|
| Br | -6.41810000 | 2.30550000 | -0.59000000 |
| C | 0.13000000 | -6.12450000 | 0.12850000 |
| C | -0.01140000 | -4.84250000 | 0.67830000 |
| C | 1.00830000 | -3.92630000 | 0.42230000 |
| C | 2.12990000 | -4.24240000 | -0.34750000 |
| C | 2.26190000 | -5.51750000 | -0.88720000 |
| C | 1.25070000 | -6.46100000 | -0.64340000 |
| C | 3.03900000 | -3.02850000 | -0.43540000 |
| C | 2.24720000 | -1.99850000 | 0.38810000 |
| N | 1.12190000 | -2.57890000 | 0.85590000 |
| C | 3.20150000 | -2.58760000 | -1.91290000 |
| C | 4.41160000 | -3.34160000 | 0.21710000 |
| C | 0.10050000 | -1.93490000 | 1.69040000 |
| C | -0.97080000 | -1.22470000 | 0.84020000 |
| C | -2.02280000 | -0.61850000 | 1.77020000 |
| N | -3.15610000 | 0.13410000 | 1.08370000 |
| C | -4.09420000 | 0.63530000 | 2.15970000 |
| C | -3.93580000 | -0.76550000 | 0.15360000 |
| C | -2.64130000 | 1.32320000 | 0.30730000 |
| O | 2.10140000 | 1.98660000 | 1.77960000 |
| C | 3.20770000 | 2.31050000 | 1.27340000 |
| C | 4.04530000 | 1.37450000 | 0.49070000 |
| C | 3.69070000 | 0.01460000 | 0.24050000 |
| C | 2.55190000 | -0.64830000 | 0.66610000 |
| C | 5.26780000 | 1.82640000 | -0.04870000 |
| C | 5.70730000 | 3.13040000 | 0.14050000 |
| C | 4.92620000 | 4.05780000 | 0.89630000 |
| C | 3.73520000 | 3.65960000 | 1.43440000 |
| N | 6.95300000 | 3.54780000 | -0.42600000 |
| O | 7.62890000 | 2.73070000 | -1.09010000 |
| O | 7.33110000 | 4.72590000 | -0.23890000 |
| H | -0.64490000 | -6.86490000 | 0.30600000 |
| H | -0.88580000 | -4.59520000 | 1.27130000 |
| H | 3.12690000 | -5.78750000 | -1.48730000 |
| H | 1.33700000 | -7.46150000 | -1.05800000 |
| H | 3.64660000 | -3.40730000 | -2.48360000 |
| H | 3.85410000 | -1.71760000 | -2.00990000 |
| H | 2.23360000 | -2.34580000 | -2.36110000 |
| H | 4.29190000 | -3.64020000 | 1.26250000 |
| H | 5.08600000 | -2.48370000 | 0.17880000 |
| H | 4.88490000 | -4.16680000 | -0.32260000 |
| H | 0.59250000 | -1.23720000 | 2.36890000 |
| H | -0.34910000 | -2.71040000 | 2.31260000 |
| H | -1.42030000 | -1.95190000 | 0.16030000 |
| H | -0.48570000 | -0.45460000 | 0.23610000 |
| H | -1.56310000 | 0.09550000 | 2.45710000 |
| H | -2.50100000 | -1.39960000 | 2.36520000 |
| H | -4.90280000 | 1.18540000 | 1.67650000 |
| H | -3.53890000 | 1.28740000 | 2.83360000 |
| H | -4.48880000 | -0.22030000 | 2.70750000 |
| H | -3.30530000 | -1.05680000 | -0.68450000 |
| H | -4.79680000 | -0.20330000 | -0.21020000 |
| H | -4.26020000 | -1.64750000 | 0.70620000 |
| H | -2.05810000 | 1.95600000 | 0.97650000 |
| H | -3.50370000 | 1.86830000 | -0.07960000 |
| H | -2.02170000 | 0.97710000 | -0.51770000 |
| H | 4.41760000 | -0.53240000 | -0.35160000 |
| H | 1.85130000 | -0.06890000 | 1.25190000 |
| H | 5.88490000 | 1.14460000 | -0.62530000 |
| H | 5.29220000 | 5.06980000 | 1.03200000 |
| H | 3.12950000 | 4.35390000 | 2.01090000 |

**SP (ethanol)**

| | | | |
|---|---|---|---|
| C | -0.77014600 | -5.44066300 | -0.72571900 |
| C | -0.41027500 | -4.08731200 | -0.85243100 |
| C | -1.25748500 | -3.12543100 | -0.29227900 |
| C | -2.43740200 | -3.49924700 | 0.37897100 |
| C | -2.77927600 | -4.83909200 | 0.50241900 |
| C | -1.94006000 | -5.82140400 | -0.06011700 |
| C | -3.10687000 | -2.25393800 | 0.94372600 |
| C | -2.40306200 | -1.12337700 | 0.07635600 |
| N | -1.11370200 | -1.73495000 | -0.28611500 |
| C | -2.71844000 | -2.11599400 | 2.43613300 |
| C | -4.63816000 | -2.24235400 | 0.81899300 |
| C | -0.15264900 | -1.03949900 | -1.13333500 |
| C | 1.20197700 | -0.85239300 | -0.41779900 |
| C | 2.18002200 | -0.12862400 | -1.34242500 |
| N | 3.57619500 | 0.11688600 | -0.77664200 |
| C | 4.38572000 | 0.83510500 | -1.83211600 |
| C | 4.27389300 | -1.17973900 | -0.44390400 |
| C | 3.52328800 | 0.98378100 | 0.45787600 |
| O | -2.12120400 | 0.02470500 | 0.92130700 |
| C | -2.46028100 | 1.29459200 | 0.59962800 |
| C | -3.16635000 | 1.61317600 | -0.58368600 |
| C | -3.50925900 | 0.51641300 | -1.47810800 |
| C | -3.17375100 | -0.74360800 | -1.16243000 |
| C | -3.48316700 | 2.94799700 | -0.84074600 |
| C | -3.10002600 | 3.93839800 | 0.06986400 |
| C | -2.39657300 | 3.62338900 | 1.24280000 |
| C | -2.07649300 | 2.29755400 | 1.50389700 |
| N | -3.43564800 | 5.32393000 | -0.20733200 |
| O | -4.06465300 | 5.58637800 | -1.24701000 |
| O | -3.08076400 | 6.19455100 | 0.60588400 |
| H | -0.12400800 | -6.20076000 | -1.15848500 |
| H | 0.50174000 | -3.81299600 | -1.37467300 |
| H | -3.68822800 | -5.13418600 | 1.02220800 |
| H | -2.20309500 | -6.87267600 | 0.02127000 |
| H | -1.63416600 | -2.06978600 | 2.57031700 |
| H | -3.09335200 | -2.98901000 | 2.97936600 |
| H | -3.15971100 | -1.21909100 | 2.87987500 |
| H | -4.97667100 | -2.44011100 | -0.20117800 |
| H | -5.04730200 | -1.27737100 | 1.13719200 |
| H | -5.06793000 | -3.01209500 | 1.46793000 |
| H | -0.56403100 | -0.06158300 | -1.39669100 |
| H | -0.01476300 | -1.58403900 | -2.07657000 |
| H | 1.58665500 | -1.83428600 | -0.13075700 |
| H | 1.02324400 | -0.28320400 | 0.49770000 |
| H | 1.79414600 | 0.85730300 | -1.61279000 |
| H | 2.33061800 | -0.69650600 | -2.26343300 |
| H | 5.38683800 | 1.00951900 | -1.43589900 |
| H | 3.89844700 | 1.78175800 | -2.06540000 |
| H | 4.43295900 | 0.20947900 | -2.72349200 |
| H | 3.74788700 | -1.67719800 | 0.36858700 |
| H | 5.29308000 | -0.94211200 | -0.13513800 |
| H | 4.27994600 | -1.81260600 | -1.33158600 |
| H | 2.98925200 | 1.90338000 | 0.21800900 |
| H | 4.54893200 | 1.20237000 | 0.75870300 |
| H | 3.00959500 | 0.44796900 | 1.25378200 |
| H | -4.03382100 | 0.73716300 | -2.40455700 |
| H | -3.40887700 | -1.56792300 | -1.82991300 |
| H | -4.02243300 | 3.21867200 | -1.74188100 |
| H | -2.11015000 | 4.40870100 | 1.93225100 |
| H | -1.53309300 | 2.02195900 | 2.40232100 |
| Br | 7.52263700 | 0.85590700 | 0.68082000 |

**MC (dmso)**

| | | | |
|---|---|---|---|
| Br | -6.41810000 | 2.30550000 | -0.59000000 |
| C | 0.13000000 | -6.12450000 | 0.12850000 |
| C | -0.01140000 | -4.84250000 | 0.67830000 |
| C | 1.00830000 | -3.92630000 | 0.42230000 |
| C | 2.12990000 | -4.24240000 | -0.34750000 |
| C | 2.26190000 | -5.51750000 | -0.88720000 |
| C | 1.25070000 | -6.46100000 | -0.64340000 |
| C | 3.03900000 | -3.02850000 | -0.43540000 |
| C | 2.24720000 | -1.99850000 | 0.38810000 |
| N | 1.12190000 | -2.57890000 | 0.85590000 |
| C | 3.20150000 | -2.58760000 | -1.91290000 |
| C | 4.41160000 | -3.34160000 | 0.21710000 |
| C | 0.10050000 | -1.93490000 | 1.69040000 |
| C | -0.97080000 | -1.22470000 | 0.84020000 |
| C | -2.02280000 | -0.61850000 | 1.77020000 |
| N | -3.15610000 | 0.13410000 | 1.08370000 |
| C | -4.09420000 | 0.63530000 | 2.15970000 |
| C | -3.93580000 | -0.76550000 | 0.15360000 |
| C | -2.64130000 | 1.32320000 | 0.30730000 |
| O | 2.10140000 | 1.98660000 | 1.77960000 |
| C | 3.20770000 | 2.31050000 | 1.27340000 |
| C | 4.04530000 | 1.37450000 | 0.49070000 |
| C | 3.69070000 | 0.01460000 | 0.24050000 |
| C | 2.55190000 | -0.64830000 | 0.66610000 |
| C | 5.26780000 | 1.82640000 | -0.04870000 |
| C | 5.70730000 | 3.13040000 | 0.14050000 |
| C | 4.92620000 | 4.05780000 | 0.89630000 |
| C | 3.73520000 | 3.65960000 | 1.43440000 |
| N | 6.95300000 | 3.54780000 | -0.42600000 |
| O | 7.62890000 | 2.73070000 | -1.09010000 |
| O | 7.33110000 | 4.72590000 | -0.23890000 |
| H | -0.64490000 | -6.86490000 | 0.30600000 |
| H | -0.88580000 | -4.59520000 | 1.27130000 |
| H | 3.12690000 | -5.78750000 | -1.48730000 |
| H | 1.33700000 | -7.46150000 | -1.05800000 |
| H | 3.64660000 | -3.40730000 | -2.48360000 |
| H | 3.85410000 | -1.71760000 | -2.00990000 |
| H | 2.23360000 | -2.34580000 | -2.36110000 |
| H | 4.29190000 | -3.64020000 | 1.26250000 |
| H | 5.08600000 | -2.48370000 | 0.17880000 |
| H | 4.88490000 | -4.16680000 | -0.32260000 |
| H | 0.59250000 | -1.23720000 | 2.36890000 |
| H | -0.34910000 | -2.71040000 | 2.31260000 |
| H | -1.42030000 | -1.95190000 | 0.16030000 |
| H | -0.48570000 | -0.45460000 | 0.23610000 |
| H | -1.56310000 | 0.09550000 | 2.45710000 |
| H | -2.50100000 | -1.39960000 | 2.36520000 |
| H | -4.90280000 | 1.18540000 | 1.67650000 |
| H | -3.53890000 | 1.28740000 | 2.83360000 |
| H | -4.48880000 | -0.22030000 | 2.70750000 |
| H | -3.30530000 | -1.05680000 | -0.68450000 |
| H | -4.79680000 | -0.20330000 | -0.21020000 |
| H | -4.26020000 | -1.64750000 | 0.70620000 |
| H | -2.05810000 | 1.95600000 | 0.97650000 |
| H | -3.50370000 | 1.86830000 | -0.07960000 |
| H | -2.02170000 | 0.97710000 | -0.51770000 |
| H | 4.41760000 | -0.53240000 | -0.35160000 |
| H | 1.85130000 | -0.06890000 | 1.25190000 |
| H | 5.88490000 | 1.14460000 | -0.62530000 |
| H | 5.29220000 | 5.06980000 | 1.03200000 |
| H | 3.12950000 | 4.35390000 | 2.01090000 |

**SP (dmso)**

| | | | |
|---|---|---|---|
| C | -1.01976100 | -5.36186500 | -0.81474000 |
| C | -0.59967700 | -4.03243200 | -0.89229700 |
| C | -1.39362500 | -3.06196300 | -0.29838600 |
| C | -2.57891000 | -3.40309300 | 0.36054800 |
| C | -2.98101600 | -4.71856000 | 0.43351900 |
| C | -2.19519200 | -5.70936400 | -0.16496000 |
| C | -3.18162300 | -2.15220200 | 0.96788300 |
| C | -2.45261800 | -1.04191300 | 0.12230600 |
| N | -1.19203500 | -1.68625200 | -0.24930600 |
| C | -2.75456200 | -2.07004000 | 2.44216700 |
| C | -4.70269000 | -2.07163600 | 0.87999700 |
| C | -0.22379500 | -1.01285200 | -1.09042500 |
| C | 1.13584400 | -0.89134300 | -0.39672600 |
| C | 2.12126900 | -0.19336200 | -1.31717800 |
| N | 3.51275500 | 0.00182200 | -0.76085400 |
| C | 4.33408600 | 0.69427800 | -1.80382900 |
| C | 4.16182600 | -1.30739600 | -0.44211700 |
| C | 3.49197700 | 0.85287000 | 0.46895500 |
| O | -2.15376000 | 0.07895400 | 0.96953800 |
| C | -2.38124800 | 1.35970300 | 0.62591100 |
| C | -3.04282000 | 1.70742600 | -0.55935000 |
| C | -3.46528700 | 0.62655600 | -1.43558500 |
| C | -3.21178600 | -0.63601800 | -1.10954100 |
| C | -3.25264900 | 3.04846400 | -0.83999800 |
| C | -2.80840800 | 4.01050000 | 0.05329400 |
| C | -2.15210700 | 3.66894900 | 1.23105600 |
| C | -1.93817900 | 2.33647900 | 1.51550200 |
| N | -3.03486700 | 5.41494700 | -0.25040300 |
| O | -3.61668900 | 5.69850900 | -1.28593900 |
| O | -2.63324500 | 6.25148200 | 0.54331300 |
| H | -0.41279300 | -6.13305000 | -1.27663700 |
| H | 0.32091100 | -3.78060900 | -1.40536200 |
| H | -3.89971800 | -4.98906900 | 0.94394300 |
| H | -2.50636400 | -6.74674700 | -0.12199300 |
| H | -1.66923500 | -2.07700900 | 2.54991100 |
| H | -3.15473900 | -2.93415900 | 2.97567500 |
| H | -3.13916700 | -1.16682100 | 2.91668500 |
| H | -5.07320500 | -2.22743100 | -0.13313400 |
| H | -5.06131200 | -1.10055000 | 1.22939600 |
| H | -5.15290900 | -2.83419800 | 1.51846800 |
| H | -0.60448100 | -0.01867600 | -1.32994400 |
| H | -0.12187000 | -1.53982900 | -2.04583700 |
| H | 1.48635400 | -1.88945000 | -0.12993500 |
| H | 0.99296800 | -0.33466600 | 0.53004300 |
| H | 1.76393200 | 0.80320100 | -1.57797400 |
| H | 2.24749500 | -0.75387900 | -2.24354100 |
| H | 5.33835800 | 0.83694200 | -1.41129100 |
| H | 3.87754600 | 1.65419800 | -2.03170600 |
| H | 4.36210500 | 0.07468400 | -2.69674300 |
| H | 3.62470500 | -1.79238300 | 0.36668000 |
| H | 5.18659600 | -1.10867100 | -0.13580900 |
| H | 4.14618400 | -1.93350400 | -1.33098700 |
| H | 2.99143800 | 1.79037100 | 0.23913500 |
| H | 4.52152100 | 1.03542500 | 0.76904800 |
| H | 2.96490200 | 0.33179300 | 1.26193700 |
| H | -3.98256300 | 0.87357300 | -2.35616300 |
| H | -3.50411000 | -1.45175200 | -1.76047200 |
| H | -3.75984500 | 3.34312500 | -1.74895900 |
| H | -1.81668900 | 4.44038700 | 1.90959500 |
| H | -1.43145600 | 2.03441500 | 2.42318600 |
| Br | 7.43542100 | 0.61148700 | 0.67129700 |

**MC (chloroform)**

| | | | |
|---|---|---|---|
| Br | -6.41810000 | 2.30550000 | -0.59000000 |
| C | 0.13000000 | -6.12450000 | 0.12850000 |
| C | -0.01140000 | -4.84250000 | 0.67830000 |
| C | 1.00830000 | -3.92630000 | 0.42230000 |
| C | 2.12990000 | -4.24240000 | -0.34750000 |
| C | 2.26190000 | -5.51750000 | -0.88720000 |
| C | 1.25070000 | -6.46100000 | -0.64340000 |
| C | 3.03900000 | -3.02850000 | -0.43540000 |
| C | 2.24720000 | -1.99850000 | 0.38810000 |
| N | 1.12190000 | -2.57890000 | 0.85590000 |
| C | 3.20150000 | -2.58760000 | -1.91290000 |
| C | 4.41160000 | -3.34160000 | 0.21710000 |
| C | 0.10050000 | -1.93490000 | 1.69040000 |
| C | -0.97080000 | -1.22470000 | 0.84020000 |
| C | -2.02280000 | -0.61850000 | 1.77020000 |
| N | -3.15610000 | 0.13410000 | 1.08370000 |
| C | -4.09420000 | 0.63530000 | 2.15970000 |
| C | -3.93580000 | -0.76550000 | 0.15360000 |
| C | -2.64130000 | 1.32320000 | 0.30730000 |
| O | 2.10140000 | 1.98660000 | 1.77960000 |
| C | 3.20770000 | 2.31050000 | 1.27340000 |
| C | 4.04530000 | 1.37450000 | 0.49070000 |
| C | 3.69070000 | 0.01460000 | 0.24050000 |
| C | 2.55190000 | -0.64830000 | 0.66610000 |
| C | 5.26780000 | 1.82640000 | -0.04870000 |
| C | 5.70730000 | 3.13040000 | 0.14050000 |
| C | 4.92620000 | 4.05780000 | 0.89630000 |
| C | 3.73520000 | 3.65960000 | 1.43440000 |
| N | 6.95300000 | 3.54780000 | -0.42600000 |
| O | 7.62890000 | 2.73070000 | -1.09010000 |
| O | 7.33110000 | 4.72590000 | -0.23890000 |
| H | -0.64490000 | -6.86490000 | 0.30600000 |
| H | -0.88580000 | -4.59520000 | 1.27130000 |
| H | 3.12690000 | -5.78750000 | -1.48730000 |
| H | 1.33700000 | -7.46150000 | -1.05800000 |
| H | 3.64660000 | -3.40730000 | -2.48360000 |
| H | 3.85410000 | -1.71760000 | -2.00990000 |
| H | 2.23360000 | -2.34580000 | -2.36110000 |
| H | 4.29190000 | -3.64020000 | 1.26250000 |
| H | 5.08600000 | -2.48370000 | 0.17880000 |
| H | 4.88490000 | -4.16680000 | -0.32260000 |
| H | 0.59250000 | -1.23720000 | 2.36890000 |
| H | -0.34910000 | -2.71040000 | 2.31260000 |
| H | -1.42030000 | -1.95190000 | 0.16030000 |
| H | -0.48570000 | -0.45460000 | 0.23610000 |
| H | -1.56310000 | 0.09550000 | 2.45710000 |
| H | -2.50100000 | -1.39960000 | 2.36520000 |
| H | -4.90280000 | 1.18540000 | 1.67650000 |
| H | -3.53890000 | 1.28740000 | 2.83360000 |
| H | -4.48880000 | -0.22030000 | 2.70750000 |
| H | -3.30530000 | -1.05680000 | -0.68450000 |
| H | -4.79680000 | -0.20330000 | -0.21020000 |
| H | -4.26020000 | -1.64750000 | 0.70620000 |
| H | -2.05810000 | 1.95600000 | 0.97650000 |
| H | -3.50370000 | 1.86830000 | -0.07960000 |
| H | -2.02170000 | 0.97710000 | -0.51770000 |
| H | 4.41760000 | -0.53240000 | -0.35160000 |
| H | 1.85130000 | -0.06890000 | 1.25190000 |
| H | 5.88490000 | 1.14460000 | -0.62530000 |
| H | 5.29220000 | 5.06980000 | 1.03200000 |
| H | 3.12950000 | 4.35390000 | 2.01090000 |

**SP (chloroform)**

| | | | |
|---|---|---|---|
| C | -0.76436800 | -5.34172300 | -0.84762800 |
| C | -0.40511000 | -3.99354800 | -0.90310800 |
| C | -1.24714100 | -3.06902400 | -0.30322800 |
| C | -2.42209800 | -3.47231700 | 0.33799600 |
| C | -2.76403100 | -4.80559000 | 0.38943000 |
| C | -1.92799400 | -5.75087800 | -0.21375900 |
| C | -3.08810600 | -2.25814400 | 0.95361800 |
| C | -2.40610700 | -1.10661200 | 0.12528900 |
| N | -1.10878100 | -1.68500800 | -0.23209300 |
| C | -2.67605600 | -2.17086300 | 2.43184700 |
| C | -4.61045600 | -2.24715500 | 0.85395100 |
| C | -0.16647300 | -0.95966300 | -1.05775400 |
| C | 1.19142600 | -0.81539400 | -0.36476800 |
| C | 2.15952300 | -0.07784400 | -1.27292700 |
| N | 3.54704500 | 0.13559600 | -0.71885200 |
| C | 4.35383200 | 0.86058300 | -1.75218100 |
| C | 4.22381000 | -1.16576000 | -0.42188800 |
| C | 3.51516900 | 0.96758400 | 0.52464900 |
| O | -2.17892600 | 0.02209100 | 0.98217800 |
| C | -2.45163300 | 1.29402700 | 0.63335300 |
| C | -3.10538600 | 1.61471800 | -0.56327400 |
| C | -3.46931200 | 0.51723500 | -1.44531200 |
| C | -3.16701900 | -0.73382600 | -1.11609500 |
| C | -3.36476400 | 2.94672400 | -0.84860500 |
| C | -2.97635500 | 3.92543000 | 0.05117900 |
| C | -2.32920000 | 3.61108300 | 1.24061500 |
| C | -2.06605400 | 2.28785300 | 1.53012000 |
| N | -3.25449600 | 5.32247200 | -0.25901900 |
| O | -3.82678600 | 5.57780900 | -1.30576800 |
| O | -2.90125200 | 6.17137400 | 0.54226300 |
| H | -0.11896900 | -6.07822700 | -1.31380900 |
| H | 0.50731900 | -3.69334100 | -1.40476800 |
| H | -3.67425500 | -5.12473600 | 0.88666500 |
| H | -2.19136400 | -6.80184800 | -0.18713200 |
| H | -1.59262600 | -2.12493500 | 2.54761400 |
| H | -3.03549000 | -3.05982400 | 2.95328400 |
| H | -3.10838000 | -1.29323600 | 2.91323100 |
| H | -4.96595800 | -2.41302000 | -0.16298500 |
| H | -5.01647400 | -1.29609500 | 1.20672900 |
| H | -5.03057800 | -3.03370700 | 1.48375700 |
| H | -0.57844900 | 0.02896200 | -1.26719500 |
| H | -0.05100800 | -1.45608000 | -2.02873100 |
| H | 1.56718000 | -1.80866300 | -0.11516900 |
| H | 1.03637000 | -0.28016300 | 0.57269400 |
| H | 1.77920100 | 0.91556200 | -1.51410100 |
| H | 2.29304200 | -0.61728100 | -2.21116100 |
| H | 5.35512100 | 1.01791600 | -1.35596400 |
| H | 3.87570300 | 1.81366400 | -1.96573900 |
| H | 4.39381000 | 0.25410400 | -2.65406200 |
| H | 3.69876200 | -1.67268300 | 0.38174000 |
| H | 5.24573900 | -0.94887200 | -0.11606400 |
| H | 4.21579100 | -1.77800900 | -1.32096400 |
| H | 3.00008400 | 1.90091000 | 0.30843300 |
| H | 4.54349300 | 1.15878500 | 0.82572100 |
| H | 2.99561600 | 0.42582400 | 1.30895800 |
| H | -3.98511700 | 0.74264900 | -2.37240900 |
| H | -3.41550600 | -1.56140900 | -1.77034200 |
| H | -3.86770700 | 3.22268700 | -1.76591300 |
| H | -2.03986900 | 4.39704700 | 1.92388700 |
| H | -1.56614500 | 2.00619400 | 2.44821100 |
| Br | 7.32809800 | 0.74956700 | 0.64905200 |

**MC (acetonitrile)**

| | | | |
|---|---|---|---|
| Br | -6.41810000 | 2.30550000 | -0.59000000 |
| C | 0.13000000 | -6.12450000 | 0.12850000 |
| C | -0.01140000 | -4.84250000 | 0.67830000 |
| C | 1.00830000 | -3.92630000 | 0.42230000 |
| C | 2.12990000 | -4.24240000 | -0.34750000 |
| C | 2.26190000 | -5.51750000 | -0.88720000 |
| C | 1.25070000 | -6.46100000 | -0.64340000 |
| C | 3.03900000 | -3.02850000 | -0.43540000 |
| C | 2.24720000 | -1.99850000 | 0.38810000 |
| N | 1.12190000 | -2.57890000 | 0.85590000 |
| C | 3.20150000 | -2.58760000 | -1.91290000 |
| C | 4.41160000 | -3.34160000 | 0.21710000 |
| C | 0.10050000 | -1.93490000 | 1.69040000 |
| C | -0.97080000 | -1.22470000 | 0.84020000 |
| C | -2.02280000 | -0.61850000 | 1.77020000 |
| N | -3.15610000 | 0.13410000 | 1.08370000 |
| C | -4.09420000 | 0.63530000 | 2.15970000 |
| C | -3.93580000 | -0.76550000 | 0.15360000 |
| C | -2.64130000 | 1.32320000 | 0.30730000 |
| O | 2.10140000 | 1.98660000 | 1.77960000 |
| C | 3.20770000 | 2.31050000 | 1.27340000 |
| C | 4.04530000 | 1.37450000 | 0.49070000 |
| C | 3.69070000 | 0.01460000 | 0.24050000 |
| C | 2.55190000 | -0.64830000 | 0.66610000 |
| C | 5.26780000 | 1.82640000 | -0.04870000 |
| C | 5.70730000 | 3.13040000 | 0.14050000 |
| C | 4.92620000 | 4.05780000 | 0.89630000 |
| C | 3.73520000 | 3.65960000 | 1.43440000 |
| N | 6.95300000 | 3.54780000 | -0.42600000 |
| O | 7.62890000 | 2.73070000 | -1.09010000 |
| O | 7.33110000 | 4.72590000 | -0.23890000 |
| H | -0.64490000 | -6.86490000 | 0.30600000 |
| H | -0.88580000 | -4.59520000 | 1.27130000 |
| H | 3.12690000 | -5.78750000 | -1.48730000 |
| H | 1.33700000 | -7.46150000 | -1.05800000 |
| H | 3.64660000 | -3.40730000 | -2.48360000 |
| H | 3.85410000 | -1.71760000 | -2.00990000 |
| H | 2.23360000 | -2.34580000 | -2.36110000 |
| H | 4.29190000 | -3.64020000 | 1.26250000 |
| H | 5.08600000 | -2.48370000 | 0.17880000 |
| H | 4.88490000 | -4.16680000 | -0.32260000 |
| H | 0.59250000 | -1.23720000 | 2.36890000 |
| H | -0.34910000 | -2.71040000 | 2.31260000 |
| H | -1.42030000 | -1.95190000 | 0.16030000 |
| H | -0.48570000 | -0.45460000 | 0.23610000 |
| H | -1.56310000 | 0.09550000 | 2.45710000 |
| H | -2.50100000 | -1.39960000 | 2.36520000 |
| H | -4.90280000 | 1.18540000 | 1.67650000 |
| H | -3.53890000 | 1.28740000 | 2.83360000 |
| H | -4.48880000 | -0.22030000 | 2.70750000 |
| H | -3.30530000 | -1.05680000 | -0.68450000 |
| H | -4.79680000 | -0.20330000 | -0.21020000 |
| H | -4.26020000 | -1.64750000 | 0.70620000 |
| H | -2.05810000 | 1.95600000 | 0.97650000 |
| H | -3.50370000 | 1.86830000 | -0.07960000 |
| H | -2.02170000 | 0.97710000 | -0.51770000 |
| H | 4.41760000 | -0.53240000 | -0.35160000 |
| H | 1.85130000 | -0.06890000 | 1.25190000 |
| H | 5.88490000 | 1.14460000 | -0.62530000 |
| H | 5.29220000 | 5.06980000 | 1.03200000 |
| H | 3.12950000 | 4.35390000 | 2.01090000 |

**SP (acetonitrile)**

| | | | |
|---|---|---|---|
| C | -1.01793700 | -5.36153900 | -0.81632600 |
| C | -0.59811500 | -4.03198900 | -0.89325100 |
| C | -1.39221100 | -3.06196900 | -0.29884700 |
| C | -2.57738300 | -3.40360500 | 0.35999800 |
| C | -2.97924200 | -4.71918400 | 0.43232800 |
| C | -2.19326700 | -5.70953900 | -0.16666000 |
| C | -3.18022600 | -2.15308800 | 0.96800800 |
| C | -2.45176200 | -1.04236700 | 0.12263400 |
| N | -1.19085300 | -1.68618500 | -0.24906200 |
| C | -2.75268000 | -2.07137300 | 2.44218300 |
| C | -4.70132900 | -2.07277600 | 0.88062800 |
| C | -0.22295300 | -1.01221400 | -1.09006100 |
| C | 1.13671900 | -0.89068400 | -0.39642900 |
| C | 2.12185200 | -0.19130300 | -1.31613300 |
| N | 3.51325700 | 0.00364300 | -0.75977000 |
| C | 4.33438200 | 0.69770200 | -1.80186800 |
| C | 4.16288300 | -1.30573300 | -0.44272900 |
| C | 3.49242900 | 0.85313200 | 0.47114400 |
| O | -2.15342300 | 0.07852700 | 0.96993400 |
| C | -2.38097700 | 1.35925900 | 0.62613700 |
| C | -3.04240400 | 1.70679900 | -0.55924700 |
| C | -3.46481900 | 0.62577500 | -1.43533800 |
| C | -3.21113400 | -0.63673600 | -1.10918000 |
| C | -3.25223700 | 3.04780600 | -0.84010100 |
| C | -2.80818500 | 4.01002500 | 0.05306100 |
| C | -2.15208900 | 3.66861600 | 1.23097200 |
| C | -1.93810800 | 2.33619100 | 1.51564400 |
| N | -3.03466900 | 5.41444600 | -0.25090200 |
| O | -3.61559900 | 5.69770100 | -1.28697700 |
| O | -2.63389000 | 6.25096100 | 0.54318600 |
| H | -0.41085500 | -6.13240800 | -1.27859200 |
| H | 0.32242700 | -3.77975500 | -1.40621400 |
| H | -3.89786600 | -4.99012300 | 0.94265900 |
| H | -2.50423700 | -6.74699900 | -0.12416500 |
| H | -1.66731900 | -2.07823900 | 2.54956100 |
| H | -3.15253200 | -2.93572000 | 2.97556000 |
| H | -3.13723000 | -1.16836300 | 2.91714500 |
| H | -5.07215800 | -2.22848200 | -0.13240400 |
| H | -5.06000800 | -1.10180200 | 1.23027900 |
| H | -5.15125000 | -2.83548700 | 1.51912700 |
| H | -0.60393600 | -0.01803500 | -1.32913200 |
| H | -0.12099400 | -1.53876700 | -2.04572500 |
| H | 1.48769300 | -1.88889500 | -0.13062100 |
| H | 0.99364000 | -0.33505100 | 0.53093500 |
| H | 1.76411700 | 0.80545000 | -1.57572100 |
| H | 2.24809600 | -0.75064900 | -2.24321500 |
| H | 5.33859600 | 0.84007500 | -1.40905000 |
| H | 3.87755900 | 1.65780200 | -2.02846900 |
| H | 4.36256400 | 0.07926700 | -2.69559700 |
| H | 3.62577600 | -1.79211000 | 0.36525300 |
| H | 5.18747900 | -1.10686000 | -0.13588200 |
| H | 4.14773800 | -1.93056400 | -1.33252200 |
| H | 2.99160900 | 1.79080000 | 0.24255300 |
| H | 4.52202500 | 1.03548900 | 0.77122600 |
| H | 2.96559500 | 0.33089100 | 1.26353100 |
| H | -3.98226200 | 0.87264300 | -2.35586900 |
| H | -3.50349300 | -1.45259900 | -1.75994300 |
| H | -3.75932100 | 3.34240000 | -1.74915600 |
| H | -1.81688100 | 4.44022200 | 1.90943500 |
| H | -1.43155200 | 2.03426900 | 2.42347400 |
| Br | 7.43025400 | 0.61124400 | 0.67089000 |
